# VPS25 Promotes an Immunosuppressive Microenvironment in Head and Neck Squamous Cell Carcinoma

**DOI:** 10.3390/biom15030323

**Published:** 2025-02-22

**Authors:** Li-Guo Chen, Yu-Han Fang, Kui-Ming Wang, Wei Zhang, Gang Chen

**Affiliations:** 1 State Key Laboratory of Oral & Maxillofacial Reconstruction and Regeneration, Key Laboratory of Oral Biomedicine Ministry of Education, Hubei Key Laboratory of Stomatology, School & Hospital of Stomatology, Wuhan University, Wuhan 430079, China; 2021203040032@whu.edu.cn (L.-G.C.); 2020203040003@whu.edu.cn (K.-M.W.); 2 College of Life Sciences, Wuhan University, Wuhan 430072, China; 2022302041131@whu.edu.cn; 3 Department of Oral and Maxillofacial Surgery, School and Hospital of Stomatology, Wuhan University, Wuhan 430079, China; 4 TaiKang Center for Life and Medical Sciences, Wuhan University, Wuhan 430071, China; 5 Frontier Science Center for Immunology and Metabolism, Wuhan University, Wuhan 430071, China

**Keywords:** ESCRT, VPS25, HNSCC, TME, immunosuppression, immunotherapy

## Abstract

The ESCRT (endosomal sorting complex required for transport) machinery is essential for various cellular processes, yet its role in head and neck squamous cell carcinoma (HNSCC) is poorly understood. We utilized The Cancer Genome Atlas (TCGA) datasets to analyze the expression of ESCRT genes. Bulk RNA-sequencing data and HNSCC tissue microarrays (TMAs) were used to evaluate VPS25 expression and its clinical significance. Single-cell RNA sequencing of tumor tissues and *VPS25* knockdown experiments in CAL27 cells were used to investigate its biological functions. Immunohistochemistry, spatial transcriptomics, and immunotherapy datasets highlighted the involvement of VPS25 in immune suppression and its potential as a predictive biomarker. The results demonstrated significant VPS25 overexpression in HNSCC tissues, which correlated with poor clinical outcomes. It promoted tumor cell proliferation and migration while reducing immune cell infiltration in the tumor microenvironment (TME). Additionally, by upregulating PVR expression in tumor cells, VPS25 activated the immunosuppressive PVR-TIGIT signaling axis, thereby facilitating immune evasion. Furthermore, *VPS25* emerged as a potential biomarker for predicting immunotherapy response. These findings highlight VPS25 as a pivotal regulator of tumor progression and immune evasion in HNSCC and a promising target for therapeutic strategies.

## 1. Introduction

The ESCRT is a highly conserved machinery essential for membrane remodeling, influencing a variety of membrane-associated processes [1]. The ESCRT machinery consists of four primary complexes—ESCRT-0, ESCRT-I, ESCRT-II, and ESCRT-III—along with essential accessory proteins like ALIX and the AAA-ATPase VPS4. These complexes function in a coordinated manner to execute diverse biological tasks, including viral budding, cytokinesis, and multivesicular body (MVB) formation [2]. The effectiveness of the ESCRT system relies on the precise interactions among its subunits, with mutations or aberrant expression leading to compromised membrane repair and biological dysfunctions [3]. Given the close link between tumorigenesis and membrane remodeling, the ESCRT has been increasingly recognized for its role in cancer initiation, progression, and metastasis [4].

Numerous studies have investigated the roles of ESCRT subunits in tumorigenesis, yet the classification of these subunits as either pro-tumorigenic or tumor-suppressive remains controversial [5]. On the one hand, ESCRT components have been shown to promote cancer progression through their influence on cytokinesis, cell cycle regulation, and metastasis. For instance, depletion of ALIX and ESCRT-I disrupts cytokinesis, leading to polyploidy and increased susceptibility to malignant transformation [6]. TSG101, a key subunit of ESCRT-I, has been implicated in the maintenance of tumors, with its knockdown triggering cell cycle arrest and apoptosis [7,8]. Moreover, Yang et al. reported that TSG101 enhances metastatic potential in oral squamous cell carcinoma (OSCC) by modulating cell cycle regulators [9]. Conversely, certain ESCRT components have been identified as tumor suppressors. For example, CHMP1A inhibits the growth of renal cell carcinoma by modulating the PI3K/mTOR/p53 signaling pathway [10], and its loss is associated with increased tumor cell proliferation [11]. VPS25 has also been described as a tumor suppressor in *Drosophila*, where it prevents neoplastic transformation by regulating endocytic trafficking, thereby maintaining proper epithelial organization and preventing metastasis [12]. Loss of VPS25 activates pathways such as JNK, JAK/STAT, and Notch, leading to increased proliferation and tumorigenesis [13,14]. These results highlight the context-specific functions of ESCRT subunits within cancer biology.

Emerging studies indicate that the ESCRT also plays pivotal roles in shaping the tumor microenvironment (TME), a heterogeneous assembly comprising stromal cells, immune cells, and signaling factors [15,16]. Research has shown that ESCRT-mediated membrane repair enables tumor cells to withstand attacks from cytotoxic T lymphocytes (CTLs), while inhibition of ESCRT components sensitizes cancer cells to CTL-mediated lysis [17]. Additionally, loss of CHMP2A enhances chemokine secretion via the NF-κB pathway, thereby facilitating NK cell infiltration and improving the efficacy of NK cell-mediated immunotherapy [18]. However, several studies have also illuminated the tumor-suppressive roles of certain ESCRT components within the TME. For example, the knockdown of VPS36, a key subunit of ESCRT-II, results in increased secretion of extracellular vesicles (EVs), influencing paracrine cell–cell communication and the homeostasis of the TME [19]. Moreover, ESCRT components have been implicated in packaging activated STING proteins into EVs. These EVs, upon uptake by bystander cells, can trigger interferon release and strengthen anti-tumor immune responses [20,21,22,23]. Collectively, these findings illustrate the complex roles of ESCRT machinery within the TME. However, the specific contributions of various ESCRT subunits in tumors remain poorly understood, highlighting the need for further investigation.

HNSCC is among the most prevalent malignancies worldwide, characterized by a high incidence and mortality rate [24]. In this study, we focus on the role of VPS25 in HNSCC and provide several novel insights. Firstly, elevated VPS25 expression is associated with poor prognosis in HNSCC patients, underscoring its clinical significance. Secondly, scRNA-seq analyses revealed that *VPS25* overexpression drives tumor cell proliferation and migration, which was further validated through *in vitro* knockdown experiments using CAL27 cells. Moreover, VPS25^high^ cancer cells were enriched within immunosuppressive microenvironments and displayed spatial separation from tu-mor-infiltrating immune cells, indicating sophisticated interactions between VPS25^high^ cancer cells and immune compartments.Mechanically, *VPS25*^high^ cancer cells were shown to suppress anti-tumor immunity through the PVR-TIGIT axis, facilitating immune evasion. Furthermore, *VPS25* may serve as a promising biomarker for predicting immunotherapy response, highlighting its potential role in guiding treatment strategies. Collectively, these findings shed light on the pivotal contribution of VPS25 to HNSCC progression and immunosuppression, as well as its implications for clinical management, paving the way for targeted therapies and personalized medicine approaches.

## 2. Materials and Methods

### 2.1. Clinical Samples

This study was conducted in accordance with the Helsinki Declaration and received approval from the Ethics Committee at the Hospital of Stomatology, Wuhan University (No. [2019] A67). All participants provided written informed consent. The clinicopathological features of patients are summarized in Table S1.

### 2.2. Data Acquisition

The bulk RNA-sequencing datasets for HNSCC were obtained from The Cancer Genome Atlas (TCGA), as well as the GEO datasets GSE25093 and GSE25099. Public single-cell RNA-sequencing datasets for HNSCC were collected from GEO entries GSE164690, GSE181919, GSE188737, GSE195655, GSE195832, and GSE227156. Spatial transcriptomics data for HNSCC were acquired from GSE181300 and GSE208253. Additionally, tumor tissue bulk RNA-sequencing datasets related to immunotherapy were retrieved from GSE255939, GSE281729, GSE195832, and the IMvigor210 dataset. Immunotherapy-related single-cell sequencing data of tumor tissues were sourced from HRA005976.

### 2.3. scRNA-Seq Data Preprocessing

scRNA-seq data from individual tumor samples were processed by quality control, dimensionality reduction, and clustering using the Scanpy [25] package (version 1.10.1) in a Python (version 3.10.0) environment. For each sample, the following criteria were applied to filter the expression matrix: (1) cells with fewer than 200 identified genes were removed; (2) the top 2% of cells with the highest UMI counts were removed; (3) cells with mitochondrial content greater than 10% were discarded; and (4) genes detected in fewer than five cells were excluded. Cellular expression data underwent normalization through total count adjustment followed by logarithmic conversion, generating the processed expression matrix. Dimensionality reduction was performed through PCA on the standardized variable gene expression matrix, with the top 20 principal components retained for subsequent cluster analysis. Cellular population analysis was conducted through Leiden clustering methodology, complemented by UMAP dimensionality reduction for effective cluster visualization.

### 2.4. Analysis of Differentially Expressed Genes and Cell Type Annotation

Differentially expressed genes (DEGs) were identified through the ‘rank_genes_groups’ function in the Scanpy package. Differential gene expression was defined by two criteria: minimum cellular detection frequency exceeding 10% within individual clusters and a mean logarithmic fold change threshold above 0.25. Cell types were annotated by integrating classical marker genes identified in the DEGs with established biological references. The expression patterns of these markers were visualized using heatmaps, dotplots, and matrixplots. In the HNSCC TME, we identified seven major cell types, including malignant cells (*KRT5*, *KRT7*, and *KRT14*), endothelial cells (*CLDN5* and *FLT1*), fibroblasts (*COL1A1*, *DCN*, and *C1R*), T/NK cells (*CD3D*, *CD4*, and *CD8A*), B/plasma cells (*CD79A*, *MS4A1*, and *MZB1*), myeloid cells (*CD68*, *CD163*, and *LYZ*), and mast cells (*CPA3*, *TPSAB1*, and *TPSB2*).

### 2.5. Functional Enrichment Analysis

Gene set enrichment analysis (GSEA) [26] (version 4.3.3) was performed to identify enriched biological pathways using the Molecular Signatures Database (MsigDB, version 2024.1). DEGs were prioritized based on their signal-to-noise ratio. GSEA was conducted with pre-ranked gene lists and 1000 permutations. Gene sets were regarded as significantly enriched if their false discovery rate (FDR) was under 0.25 and the adjusted *p*-value was below 0.05. The results were visualized using GSEA enrichment plots to show the normalized enrichment score (NES) and gene set distributions.

### 2.6. Tumor Microenvironment Evaluation

The ESTIMATE algorithm [27] was employed to assess stromal and immune cell infiltration in the tumor microenvironment. Using the estimate R package (version 1.0.13), stromal scores, immune scores, and composite ESTIMATE scores were calculated based on predefined gene expression signatures. Normalized gene expression data served as input, and scores were standardized for cross-sample comparison. Higher stromal and immune scores indicate a greater presence of respective components, while the ESTIMATE score provides an overall measure of non-tumor cell infiltration in the tumor microenvironment.

### 2.7. Analysis of Intercellular Communication

Intercellular communication networks were analyzed through CellChat [28] (version 1.6.1), a computational tool that evaluates known receptor–ligand pairing mechanisms across distinct cellular populations. Normalized gene expression matrices and cell type annotations were used as input for the analysis. CellChat identified significant ligand–receptor interactions across cell types by statistically assessing pairwise interactions through permutation testing. Statistically significant intercellular communications were identified using a statistical significance cutoff of *p* < 0.05. Visualization was carried out using the inbuilt plotting functions from CellChat.

### 2.8. Transcription Factor Regulatory Network Analysis

The transcription factor (TF) network was generated with the pySCENIC [29] (version 0.12.1), integrating single-cell expression profiles with TF databases. Initial network inference was performed through GRNBoost2, which establishes potential regulatory associations by analyzing gene co-expression patterns. CisTarget was then utilized to refine the network by excluding indirect targets and identifying transcription factor binding motifs. Subsequently, AUCell quantified regulon activity across individual cells. Regulons with the highest regulon specificity scores (RSSs) were visualized in RStudio (version 2013.12.1).

### 2.9. Spatial Transcriptomics Data Preprocessing

Spatial transcriptomics data were preprocessed using the Scanpy package. Raw count matrices and spatial metadata were loaded into an AnnData object, followed by quality control to filter spots based on total counts (min 400, max 35,000), mitochondrial gene percentage (less than 20%), and gene detection (min 10 cells). Histograms were used to visualize data distributions and set appropriate filtering thresholds. Data normalization was performed using ‘sc.pp.normalize_total’, scaling counts per spot to a fixed total, followed by log transformation with ‘sc.pp.log1p’. Dimensionality reduction was performed with PCA, and a nearest-neighbor graph was constructed using ‘sc.pp.neighbors’. Data visualization was conducted with UMAP to identify spatially distinct clusters, providing a foundation for further analysis. Signature region scoring for tumor (*KRT14*, *KRT1*, *DMKN*, *EPCAM*, and *KRT5*), stromal (*DCN*, *COL1A1*, *COL1A2*, *MME*, and *PECAM1*), and immune (*PTPRC*) regions was performed using the ‘sc.tl.score_genes’ function in Scanpy with default parameters.

### 2.10. Spatial Transcriptomics Deconvolution

The Tangram [30] algorithm (version 1.0.4) was used for the deconvolution of spatial transcriptomics data to estimate cell type proportion and distribution. The method involved using the spatially resolved gene expression data as input, combined with a reference scRNA-seq dataset of known cell types. First, the spatial expression data were preprocessed as described previously. The Tangram algorithm was then applied to map the cell type signatures from the scRNA-seq data onto the spatial transcriptomics data. This method assigns cell type proportions to each spatial spot by maximizing the overlap between the observed expression profiles and the reference profiles. The resulting cell type estimates were visualized in the spatial context using UMAP and spatial plots to investigate tissue-specific patterns and cellular composition in different regions.

### 2.11. Spatial Distance Analysis

Spatial distance analysis was carried out with the Squidpy package [31] (version 1.6.2) to investigate the spatial relationships between cell types in the tissue. The spatial transcriptomics dataset was first preprocessed, including normalization and cell type annotation. A spatial graph was constructed using Squidpy’s ‘sq.gr.spatial_neighbors’ function, which quantifies the physical proximity between spots. To analyze preferential interactions between cell types, the ‘sq.gr.nhood_enrichment’ function was applied, which calculates neighborhood enrichment. The results were visualized using the ‘sq.pl.nhood_enrichment’ function to highlight spatial patterns and associations. Additionally, to examine co-occurrence in spatial dimensions, the ‘sq.gr.co_occurrence’ function was used. This function computes the conditional probability of cluster co-occurrence based on the original spatial coordinates and cluster annotations, with visualization performed using ‘sq.pl.co_occurrence’. These analyses provided valuable insights into the spatial organization and dynamic interactions within the TME.

### 2.12. Prediction of Immunotherapy Efficacy

To predict the efficacy of immunotherapy, we calculated the tumor immune dysfunction and exclusion (TIDE) score for each HNSCC patient based on their gene expression profiles using the TIDE database. The TIDE score is a reliable tool to assess potential response to immune checkpoint blockade (ICB) therapy [32]. Additionally, we utilized the IMvigor210 dataset, an independent immunotherapy trial, to further evaluate the predictive capability of the VPS25-related signature for immunotherapy response [33].

### 2.13. Immunohistochemistry Staining

Paraffin-embedded HNSCC tissue microarrays were processed for standard deparaffinization and rehydration, followed by antigen heat retrieval using EDTA buffer (pH 9.0) for 15 min. The slides were incubated with 3% hydrogen peroxide followed by 5% goat serum at 37 °C for 20 min. The primary antibodies used were VPS25 (1:400; 15669-1-AP; Proteintech Group, Rosemont, IL, USA), CD8 (1:5000; 66868-1-lg; Proteintech), CD20 (1:200; ab64088; Abcam, Cambridge, UK), CD11c (1:400; 45581; CSTCell Signaling Technology, Danvers, MA, USA), and CD68 (1:200; sc-59103; Santa Cruz Biotechnology, Dallas, TX, USA). Antibodies were incubated overnight at 4 °C, followed by incubation with secondary antibodies and streptavidin–biotin complexes at 37 °C for 20 min. DAB reagent was used for color development, and counterstaining was performed with hematoxylin. The VPS25 expression level and the number of tumor-infiltrating immune cells per unit area were calculated using CaseViewer software (version 2.4) and the Quant-Center image analyzer (version 2.2, 3DHISTECH, Budapest, Hungary). Staining intensity was represented by the histochemical score (H-score).

### 2.14. Construction of VPS25-Knockdown Cells

Lentiviral vectors encoding *human* *VPS25* shRNA (forward sequence 5′-3′: AGTCGATCCAGATTGTATTAGTTCAAGAGACTAATACAATCTGGATCGACTTTTTTT) or shRNA-control (shCTL) were produced in 293FT cells. After 48 h, viral particles were collected and used to transduce CAL27 cells, which were then selected with puromycin.

### 2.15. Western Blotting

Total protein lysates were extracted and transferred to a PVDF membrane. The membrane was blocked with 5% fat-free milk for 1 h at room temperature. After blocking, it was incubated overnight at 4 °C with the primary antibody (VPS25: 1:1000, 15669-1-AP, Proteintech Group, Rosemont, IL, USA; PVR: 1:1000, ab267788, Abcam, Cambridge, UK). The membrane was then incubated with HRP-conjugated secondary antibody for 1 h at room temperature. The protein signals were detected using an ECL reagent (Sigma, St. Louis, MI, USA), with β-actin as the loading control.

### 2.16. Cell Proliferation Assay

Cells were seeded into 96-well plates at 3000 cells per well. After the cells had fully adhered, CCK-8 solution was added and incubated at 37 °C for 1.5 h. Absorbance was then measured at 450 nm using a microplate reader. Cell proliferation was assessed every 12 h.

### 2.17. Colony Formation Assay

Cells were plated at a density of 3000 per well in 6-well plates. The culture medium was refreshed every 3 days, and the cells were cultured continuously for 15 days. Afterward, the plates were fixed in 4% paraformaldehyde for 1 h. The colonies were subsequently stained with 0.1% crystal violet for 15 min. After washing and drying, colony images were captured by microscope.

### 2.18. Wound Healing Assay

A uniform wound was generated in the cell monolayer using sterile technique, with a pipette tip carefully drawn across the confluent culture in six-well plates. Immediately after, images of the scratched area were captured. The cells were then cultured in serum-free medium. After 24 h, the scratched area was photographed again, and the change in wound area was measured using ImageJ (version 1.53e).

### 2.19. Cell Migration Assay

After 24 h of starvation, cells (1.0 × 10^5^) were resuspended in serum-free medium and were seeded into the transwell upper chamber, while the lower chamber contained medium with 10% serum. Following incubation at 37 °C for 36 h, the cells were fixed with 4% paraformaldehyde and stained with 0.1% crystal violet. Resident cells remaining in the upper compartment were carefully eliminated. Images of cells that migrated to the lower chamber were captured under a microscope, and the number of migrated cells was quantified using ImageJ.

### 2.20. Reverse Transcription Quantitative PCR (RT-qPCR)

Total RNA was extracted using TRIzol, and cDNA was synthesized with the HiScript II Q RT SuperMix (Vazyme, Nanjing, China). Real-time PCR was performed using ChamQ SYBR qPCR Master Mix (Vazyme) on a LightCycler^®^ 480 instrument. Gene expression levels were quantified relative to the *GAPDH* control using the 2^−ΔΔCt^ method. The following primer sequences were used (5′-3′): *VPS25*, forward, GTTTCGAGTGGCCGTGGCAGTATCGCTTCC; reverse, GGAGGTAAGAAGTAAAGGGAGACAGGTCC. *PVR*, forward, GGATATCTGGCTCCGAGTGC; reverse, CTCCACCTTGCAGGTCACAT. *GAPDH*, forward, ATCACCATCTTCCAGGAGCG; reverse, TGGACTCCACGACGTACTCA.

### 2.21. Statistical Analysis

Statistical analyses were carried out in GraphPad Prism 9.5. Bivariate comparisons were evaluated through Student’s *t*-test, while survival outcomes were assessed using Kaplan–Meier methodology with Mantel–Cox log-rank testing. Prognostic value assessment of VPS25 expression patterns was performed through Cox proportional hazards regression modeling. Statistical significance was determined at the conventional threshold of *p* < 0.05.

## 3. Results

### 3.1. The Increased Expression of VPS25 Is Associated with Poor Prognosis in HNSCC

**Figure 1:**
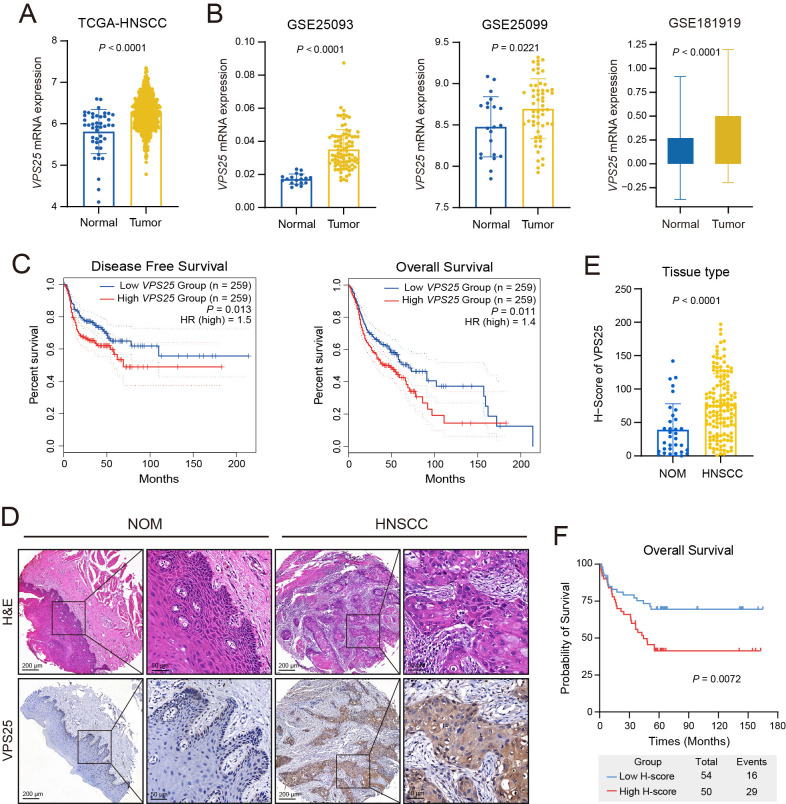
Expression pattern and prognostic value of VPS25 in HNSCC. (**A**,**B**) Comparison of *VPS25* expression levels between normal tissues and tumor tissues in the TCGA-HNSC (**A**), GSE25093, GSE25099, and GSE181919 cohorts (**B**). (**C**) Disease-free survival (DFS) and overall survival (OS) curves for HNSCC patients with high (*n* = 259) and low (n = 259) *VPS25* mRNA expression levels in the TCGA database. (**D**,**E**) Immunohistochemistry images (**D**) and quantification (**E**) of VPS25 staining in normal and tumor tissues. (**F**) Overall survival curves for HNSCC patients with different VPS25 protein expression levels. Data are presented as means ± SD for panels (**A**,**B**,**E**).

To evaluate the role of ESCRT family subunits in HNSCC development and progression, we analyzed mRNA expression profiles of these subunits in normal mucosae and tumor tissues utilizing the TCGA database. Our analysis revealed that 16 ESCRT genes were significantly upregulated in HSNCC patients (Figure S1A,B). We further examined the impact of these genes on long-term survival outcomes for HNSCC patients (Figure S2). Notably, we found that *VPS25*, a component of the ESCRT-II complex, was markedly overexpressed in HNSCC tumor tissues and correlated with poor overall survival (Figure 1A,C). Among the ESCRT family subunits, *VPS25* demonstrated the highest hazard ratio (HR) for overall survival, underscoring its potential as a prognostic marker (Figure S1C). Consequently, *VPS25* was selected for further analysis in this study.

To assess *VPS25* expression levels, we examined several publicly available RNA sequencing datasets related to HNSCC, including TCGA-HNSC, GSE25093, GSE25099, and GSE181919. Comparative analysis revealed markedly elevated *VPS25* expression levels in HNSCC specimens relative to their normal counterparts (Figure 1A,B). Further analysis revealed that elevated *VPS25* expression was associated with poorer disease-free survival and overall survival in HNSCC patients (Figure 1C). To confirm VPS25 expression at the protein level, we performed immunohistochemistry (IHC) using a tissue microarray (TMA) containing 33 normal oral mucosae (NOM) and 138 human HNSCC tissues (Table S1). The IHC results demonstrated strong cytoplasmic staining of VPS25 in HNSCC cancer cells, contrasting with minimal staining in epithelial cells of NOM (Figure 1D). Quantitative analysis confirmed significantly elevated VPS25 protein levels in HNSCC tissues compared to normal mucosae (Figure 1E).

We next explored the potential of VPS25 expression levels to stratify patients based on overall survival. Receiver operating characteristic (ROC) analysis determined an H-score threshold of 73.85 (Figure S1D), indicating that VPS25 expression levels could effectively stratify patients regarding overall survival. Comparative survival analysis revealed markedly improved clinical outcomes in cases demonstrating reduced VPS25 levels compared to their high-expression counterparts (Figure 1F and Figure S1E).

When we examined clinical variables, we found no significant differences in VPS25 protein levels across groups stratified by gender, age, clinical stage, TNM stage, or pathological grade (Figure S3A). However, univariate Cox regression analysis identified VPS25 as a robust predictor of overall survival in HNSCC, with high expression significantly correlating with poor prognosis (*p* = 0.005) (Figure S3B). Subgroup Cox regression analysis further demonstrated that elevated VPS25 levels were significantly correlated with poorer prognosis specifically in male patients, individuals under 60 years old, and those with advanced T3/T4 stages, lymph node metastasis, clinical stages III/IV, or poorly to moderately differentiated HNSCC (Figure S3C). In conclusion, our findings highlight that elevated VPS25 expression serves as a strong predictor of unfavorable clinical outcomes in HNSCC, warranting further investigation into its role in tumor progression and potential therapeutic targeting.

### 3.2. Increased Expression of VPS25 Promotes Progression of HNSCC

**Figure 2:**
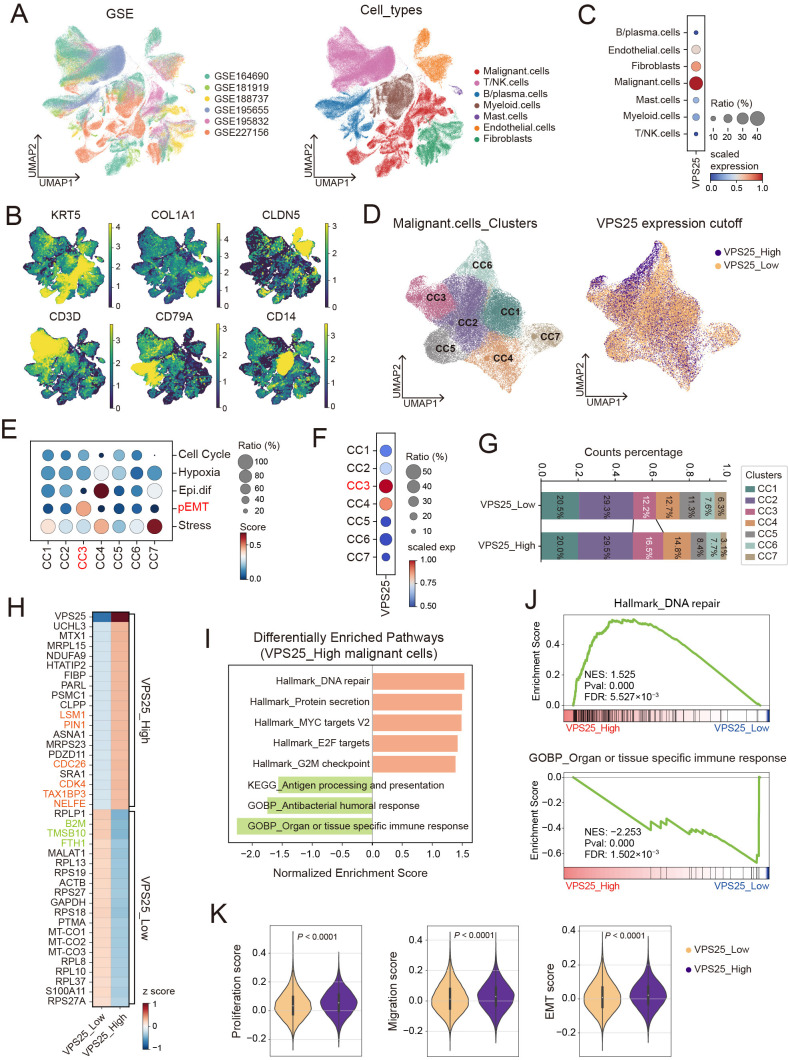
Expression patterns and functional analysis of *VPS25* in the tumor microenvironment. (**A**) UMAP plots of six publicly available HNSCC scRNA-seq datasets after processing, integration, clustering, and annotation. (**B**) UMAP embedding demonstrates the standardized expression levels of marker genes for the major cell populations in the HNSCC tumor microenvironment. (**C**) The primary cell types in the tumor microenvironment expressing *VPS25*. (**D**) Subcluster typing of HNSCC tumor cells and binary classification based on *VPS25* median expression levels. (**E**) Functional gene set scores for 7 HNSCC cancer cell subtypes. (**F**) *VPS25* expression among 7 cancer cell subtypes. (**G**) Stacked bar chart showing the proportion of functional subtypes in *VPS25*^low^ and *VPS25*^high^ tumor cells. (**H**) Heatmap of differentially expressed genes (DEGs) between *VPS25*^low^ and *VPS25*^high^ cancer cells. (**I**,**J**) Gene set enrichment analysis (GSEA) for the *VPS25*^low^ and *VPS25*^high^ cancer cells, displayed as a bar chart (**I**) and line graphs for representative pathways (**J**). (**K**) Functional scores for proliferation, migration, and invasion between *VPS25*^low^ and *VPS25*^high^ tumor cells. UMAP: Uniform manifold approximation and projection.

To investigate the role of VPS25 in HNSCC, we analyzed six publicly available scRNA-seq datasets (GSE164690, GSE181919, GSE188737, GSE195655, GSE195832, and GSE227156) derived from a total of 57 HNSCC patients (Figure 2A and Figure S4A). To accurately define and visually represent the cellular composition of the TME, we employed UMAP analysis to reduce the high-dimensional gene expression data into two dimensions, where each dot represents a single cell, and clusters represent cell populations with similar gene expression profiles. This approach provides an intuitive visualization of cellular heterogeneity within the TME, with distinct cell populations clearly separated in the visual space. After preprocessing and integrating the data, we classified the TME into seven major cell types, including malignant cells, fibroblasts, endothelial cells, myeloid cells, T/NK cells, B/plasma cells, and mast cells (Figure 2A). The expression of marker genes distinctly delineated these populations (Figure 2B and Figure S4B), and copy number variation (CNV) analysis successfully separated malignant cells from other cell types (Figure S4C). Notably, *VPS25* was predominantly expressed in malignant cells (Figure 2C), prompting us to further investigate its biological function within this subset.

We further clustered cancer cells using the Leiden graph-clustering method and performed binary classification based on the median *VPS25* expression level (Figure 2D and Figure S4D). Among the seven functional subpopulations of cancer cells, the CC3 cluster exhibited the most elevated *VPS25* expression levels (Figure 2F). Using the classification framework established by Puram et al. [34], we identified the CC3 malignant cell subgroup, characterized as a partial epithelial–mesenchymal transition (p-EMT) population with high expression of *VIM* and *TGFBI* (Figure 2E and Figure S4E,F). Remarkably, the CC3 subgroup exhibited a larger proportion of cancer cells with elevated *VPS25* expression (*VPS25*^high^) compared to those with low *VPS25* expression (*VPS25*^low^) (Figure 2G), suggesting that *VPS25*^high^ cancer cells display enhanced invasive potential.

Subsequently, we compared the molecular characteristics of *VPS25*^high^ and *VPS25*^low^ malignant cells. Differentially expressed gene (DEG) analysis indicated that *VPS25*^high^ malignant cells showed significantly increased expression of genes related to proliferation and the cell cycle, including *LSM1*, *PIN1*, *CDC26*, and *CDK4* [35,36,37,38], while *VPS25*^low^ cancer cells exhibited higher levels of immune-activating genes, including *B2M* and *FTH1* [39,40] (Figure 2H). Gene set enrichment analysis (GSEA) further revealed that genes in *VPS25*^high^ cancer cells were enriched in pathways associated with cell proliferation, including DNA repair mechanisms, MYC targets, and E2F targets. In contrast, genes in *VPS25*^low^ cells exhibited stronger enrichment in pathways related to antigen presentation and immune response (Figure 2I,J). Additionally, functional scoring using epithelial cell proliferation, migration, and epithelial–mesenchymal transition (EMT) gene sets from the MSigDB confirmed the enhanced proliferation, migration, and invasion capabilities in *VPS25*^high^ cancer cells (Figure 2K). Transcription factor analysis identified the Fos and Jun families as key regulators of *VPS25*^high^ cancer cells (Figure S4G). These transcription factors form the AP-1 complex, which is pivotal in tumorigenesis and is associated with essential malignant biological processes, including proliferation, invasion, apoptosis resistance, drug resistance, metabolic reprogramming, and immune evasion [41,42,43]. In brief, scRNA-seq analyses showed that *VPS25*^high^ cancer cells exhibit enhanced malignant biological behaviors, while *VPS25*^low^ cells display a stronger immune response.

### 3.3. Knockdown of VPS25 Suppresses the Proliferation and Migration of HNSCC Cells

**Figure 3:**
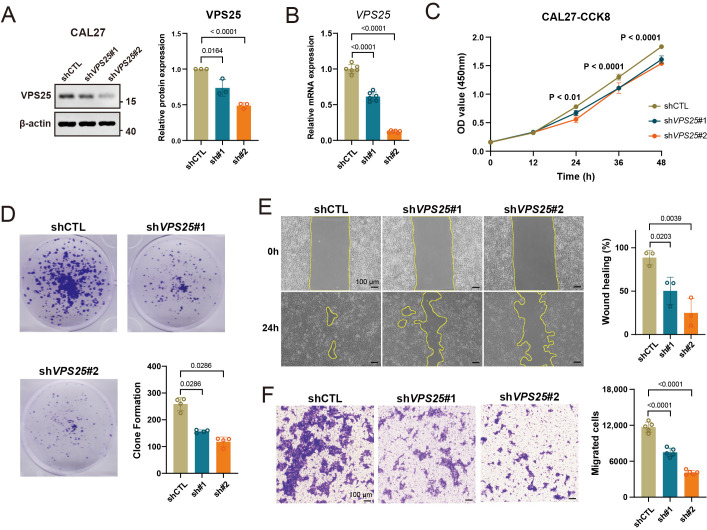
Knockdown of *VPS25* decreases proliferation and impairs migration in HNSCC cells. (**A**,**B**) WB (**A**) and qRT-PCR (**B**) experiments show the efficiency of *VPS25* KD CAL27 cells. (**C**,**D**) Proliferation analyses of CAL27 cells with *VPS25* knockdown or control cells were assessed by a cell counting kit-8 (CCK8) assay (**C**) and colony formation assay (**D**). (**E**) Illustrative micrographs and quantitative analysis of wound closure rates in shCTL and sh*VPS25* CAL27 cells at 0 h and 24 h post-wounding. (**F**) Representative images and quantification of migrated cells in shCTL and sh*VPS25* CAL27 cells. Scale bar = 100 μm. Data are presented as means ± SD. Original Western blot image of (**A**) can be found in Figure S11.

To validate the biological functions of VPS25 in HNSCC, we established *VPS25* knockdown (KD) in the human HNSCC cell line, CAL27. The successful silencing was confirmed through Western blot and qRT-PCR (Figure 3A,B). We performed cell counting kit-8 (CCK8) and colony formation assays to assess the proliferation of *VPS25* KD CAL27 cells and found that cell proliferation was significantly inhibited in *VPS25* KD CAL27 cells (Figure 3C,D). Additionally, we evaluated the migration abilities of *VPS25* KD CAL27 cells using wound healing and transwell assays, which demonstrated a marked suppression of migration in *VPS25* KD cells (Figure 3E,F). RNA sequencing of *VPS25* KD cancer cells revealed enrichment in immunoregulatory pathways, encompassing immune activation, inflammatory processes, and cytokine-mediated signaling pathways (Figure S5). In summary, our findings suggest that VPS25^high^ cancer cells not only exhibit enhanced malignant behaviors but also exhibit immune evasion, marking them as a more aggressive subgroup within HNSCC.

### 3.4. VPS25^high^ Cancer Cells Reside in an Immunosuppressive Microenvironment

**Figure 4:**
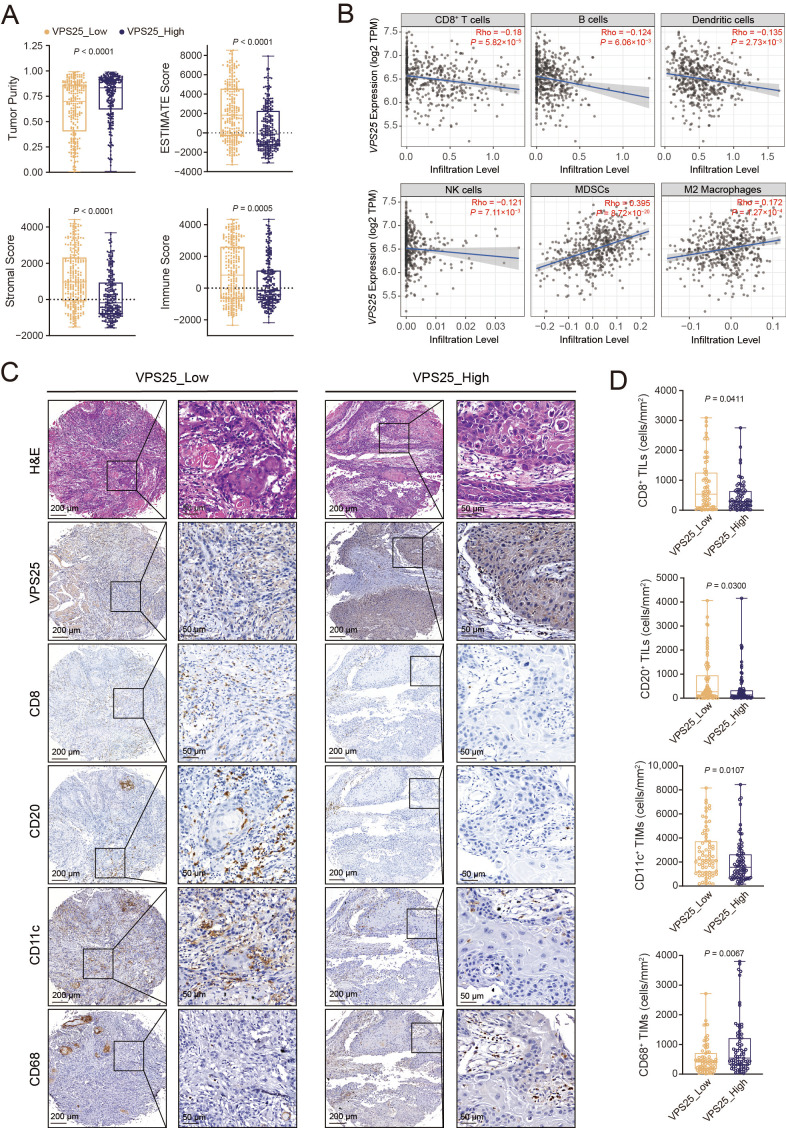
Relationship between VPS25 and tumor immune infiltration. (**A**) ESTIMATE scores were calculated for TCGA-HNSC specimens stratified by *VPS25* expression levels. (**B**) Correlation between VPS25 mRNA expression levels and tumor-infiltrating immune cell infiltration levels. (**C**) Representative immunohistochemistry images of tumor-infiltrating immune cells in HNSCC tumor samples with different VPS25 expression. (**D**) Differences in statistics in the number of tumor-infiltrating immune cells per unit area between VPS25^low^ and VPS25^high^ tumor tissue. NK cells: natural killing cells, MDSCs: myeloid-derived suppressor cells, TILs: tumor-infiltrating lymphocytes, TIMs: tumor-infiltrating myeloid cells. CD8: the marker of CD8^+^ T cells; CD20: the marker of B cells; CD11c: the marker of DCs; CD68: the marker of macrophages.

We further explored the immune microenvironment surrounding VPS25^high^ cancer cells. Using the TCGA-HNSCC dataset, patients were stratified into *VPS25*^high^ (n = 258) and *VPS25*^low^ (n = 257) groups based on their *VPS25* expression levels. ESTIMATE analysis revealed a decrease in immune infiltration in *VPS25*^high^ tumors (Figure 4A). Correlation analysis demonstrated an inverse relationship between *VPS25* levels and the presence of CD8^+^ T cells, B cells, dendritic cells (DCs), and natural killer (NK) cells. Conversely, *VPS25* expression was positively correlated with the accumulation of immunosuppressive myeloid-derived suppressor cells (MDSCs) and M2 macrophages (Figure 4B).

To further delineate the association between VPS25 and tumor-infiltrating immune cells, we performed IHC staining on our TMAs. The results showed that samples with VPS25^high^ cancer cells exhibited reduced infiltration of CD8^+^ T cells, DCs, and B cells, alongside an increased density of tumor-associated macrophages (TAMs) (Figure 4C,D). Collectively, these results indicate that high VPS25 expression in cancer cells is associated with an immunosuppressive tumor microenvironment within HNSCC, highlighting the immunosuppressive role of VPS25^high^ cancer cells.

### 3.5. Spatial Segregation of VPS25^high^ Cancer Cells from Tumor-Infiltrating Immune Cells

**Figure 5:**
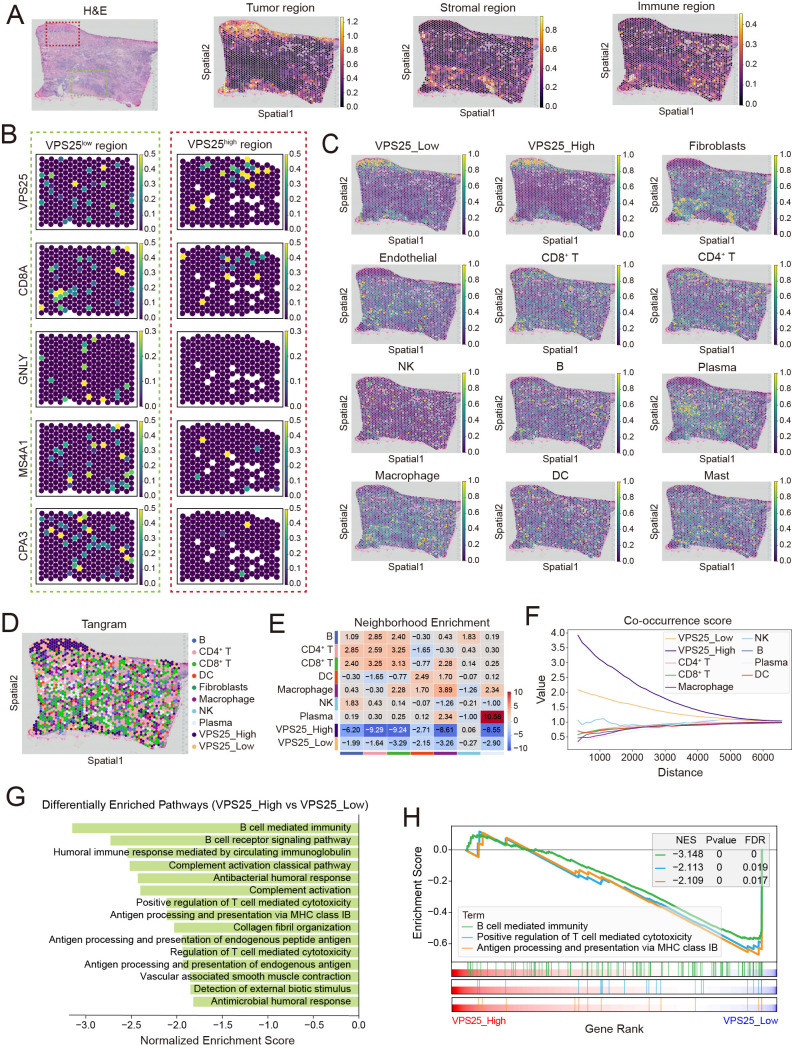
Spatial analysis of *VPS25*^high^ cancer cells and tumor-infiltrating immune cells. (**A**) Hematoxylin and eosin (HE) staining of a representative HNSCC tumor tissue with spatial transcriptomics data, showing delineation into tumor, stromal, and immune-infiltrated regions based on marker gene expression. (**B**) UMAP visualization of expression levels and spatial distribution of immune cell markers in the *VPS25* high-expression tumor region (red box area in Figure (**A**)) and the *VPS25* low-expression tumor region (green box area in Figure (**A**)). (**C**) Mapping scRNA-seq annotations to spatial transcriptomics images. (**D**) UMAP shows the dominant cell type distribution based on ranked subpopulation prediction scores in each spatial spot. (**E**) Neighbor enrichment score heatmap illustrating spatial location relationships among different cell types. (**F**) Visualization of cluster co-occurrence in spatial dimensions. (**G**,**H**) GSEA of spatial spots categorized by *VPS25*^low^ and *VPS25*^high^ cancer cells, with bar plots summarizing enriched pathways (**G**) and line plots demonstrating representative immune-related pathways (**H**). NES: normalized enrichment score, FDR: false discovery rate. *p* value < 0.05 and FDR < 0.25 indicate that the gene set is significantly enriched and the result is likely to be true rather than a false positive.

To explore the spatial relationships between VPS25^high^ cancer cells and immune infiltration, we analyzed spatial transcriptomics (ST) data from HNSCC. Histological features identified through H&E staining, combined with marker gene enrichment patterns, allowed us to categorize tissue sections into tumor, stromal, and immune-infiltrating regions (Figure 5A, Figures S7A and S8A). Additionally, scRNA-seq data from HNSCC (GSE181919) defined the TME, encompassing tumor cells, CD4^+^ and CD8^+^ T cells, NK cells, B cells, plasma cells, macrophages, DCs, mast cells, fibroblasts, and endothelial cells (Figure S6A). Marker genes effectively distinguished these subpopulations (Figure S6B).

Based on pathological features and *VPS25* gene expression, we identified the *VPS25*^high^ region (red box area in Figure 5A) and the *VPS25*^low^ region (green box area in Figure 5A) on tissue sections. A preliminary immune infiltration assessment was conducted in these two regions using immune cell marker gene expression. The results revealed that, compared to the *VPS25*^low^ region, immune cells such as CD8^+^ T cells (*CD8A*), NK cells (*GNLY*), and B cells (*MS4A1*, *CD79A*) were less infiltrated in the *VPS25*^high^ region (Figure 5B, Figures S7B and S8B). To gain a more accurate understanding of immune cell infiltration, we applied the Tangram algorithm to map the single-cell gene expression matrix onto the spatial transcriptomics profiles (Figure 5C, Figures S7C and S8C). To approximate cell type distribution, we assigned a dominant cell type to each spatial spot based on ranked subpopulation prediction scores. The spatial distribution of cell types was subsequently visualized using UMAP projections (Figure 5D, Figures S7D and S8D). Neighbor enrichment scores and cluster co-occurrence analysis indicated that *VPS25*^high^ cancer cells were significantly more distant from immune cells, particularly CD4^+^ and CD8^+^ T cells, compared to their *VPS25*^low^ counterparts (Figure 5E,F, Figures S7E,F and S8E,F).

GSEA of spatial spots further highlighted these differences. Regions with *VPS25*^high^ cancer cells showed a lack of immune activation signals and exhibited an immune-desert phenotype (Figure 5G). We primarily showcased the gene set entries for B cell-mediated immunity, T cell-mediated cytotoxicity, and antigen processing and presentation. The GSEA plot displays the enrichment score (y-axis) along the ranked list of genes (x-axis) for each gene set. Notably, the negative enrichment scores suggest that immune-related biological processes are suppressed in the *VPS25*^high^ cancer cells (Figure 5H). In contrast, regions with *VPS25*^low^ cancer cells demonstrated active antigen processing and presentation, as well as T cell-mediated cytotoxicity (Figures S7G and S8G). These results, derived from spatial transcriptomics data, confirm that *VPS25*^high^ cancer cells predominantly occupy immunosuppressive regions within the TME.

### 3.6. VPS25^high^ Cancer Cells Exhibit Immunosuppressive Effects Through the PVR-TIGIT Axis

**Figure 6:**
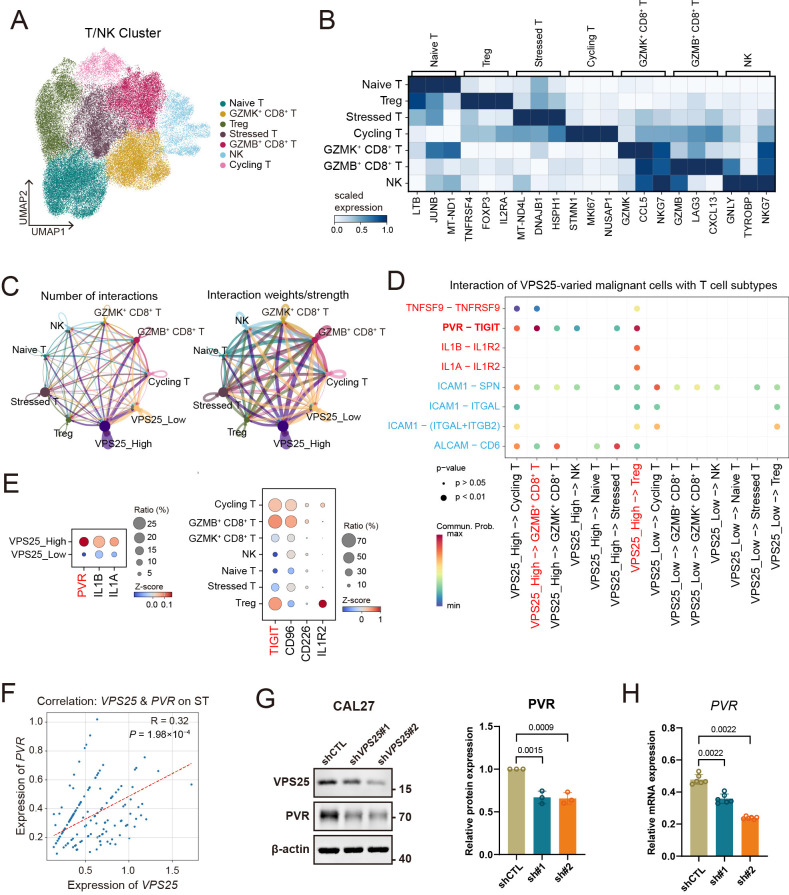
Analysis of intercellular communication between *VPS25*^high^ cancer cells and T/NK cell subsets. (**A**) UMAP embedding displaying subclusters of tumor-infiltrating T/NK cells. (**B**) Matrixplot showing marker genes for T/NK cell subgroups. (**C**) Circle plots showing the number and strength of signaling interactions between *VPS25*^low^ or *VPS25*^high^ cancer cells and T/NK cell subgroups. (**D**) Bubble plot illustrating immunosuppressive signals (red markers) and immune recruitment signals (blue markers) received by T/NK cell subgroups from tumor cells. (**E**) Expression levels of significantly different immunosuppressive signal ligand–receptor genes in cancer cells and T/NK cell subpopulations. (**F**) Correlation analysis between *PVR* and *VPS25* expression levels in tumor cell spots from the spatial transcriptomic dataset. (**G**,**H**) WB (**G**) and qRT-PCR (**H**) experiments show changes in PVR expression in *VPS25* knockdown (KD) CAL27 cells. Original Western blot image of (**G**) can be found in Figure S12.

T cells are essential for mediating anti-tumor immunity within the TME. To determine the influence of *VPS25*^high^ cancer cells on T cell function, we performed T cell subclassification in HNSCC. Based on functional characteristics and marker gene expression, T cells were categorized into seven subpopulations, including naive T cells, GZMK^+^ CD8^+^ T cells, GZMB^+^ CD8^+^ T cells, NK cells, stressed T cells, regulatory T cells (Tregs), and cycling T cells (Figure 6A,B). Among these, GZMK^+^ CD8^+^ T cells, GZMB^+^ CD8^+^ T cells, and NK cells exhibited high levels of effector cytotoxic genes such as *GZMA*, *GZMB*, *NKG7*, *PRF1*, and *GNLY*, contributing significantly to anti-tumor immunity. Conversely, Tregs, characterized by increased expression of *FOXP3* and *IL2RA*, exhibited robust immunosuppressive activity (Figure S9B).

To investigate the effects of *VPS25*^high^ cancer cells on T cell subsets, we employed CellChat to analyze the intercellular communication between *VPS25*^high^ cancer cells and various T cell subtypes. The circle plots illustrate the signaling interactions between different cell populations, where the line color represents the signals emitted by each subgroup and the line thickness indicates the number and strength of the interactions. The results show that *VPS25*^high^ cancer cells engage more strongly with T cell subpopulations than *VPS25*^low^ cancer cells (Figure 6C). To further investigate the specific signaling from *VPS25*^high^ cancer cells to T/NK cell subpopulations, we conducted a detailed analysis of the signaling emission and reception. The heatmap reveals significant differences in the signaling of ICAM, ALCAM, PVR, IL1, and CD137 emitted by *VPS25*^high^ and *VPS25*^low^ cancer cells (Figure S9C). ICAM and ALCAM, which are cell adhesion molecules, are associated with immune cell recruitment, while PVR, IL1, and CD137 are co-stimulatory signals related to immune cell activation and inhibition. Focusing on the co-stimulatory signals, we found that the PVR-TIGIT signaling axis, primarily received by GZMB^+^ CD8^+^ T cells and Tregs, is significantly upregulated in *VPS25*^high^ cancer cells (Figure 6D). Additionally, IL1 signaling is mainly mediated through IL1R2, which is specifically expressed on Tregs (Figure 6D). These pathways are well established for their immunosuppressive roles, suppressing CD8^+^ T cell cytotoxicity while concurrently enhancing Treg activation and function [44,45,46,47].

Subsequent analysis showed markedly higher *PVR* transcript expression in *VPS25*^high^ tumor cells relative to their low-expressing counterparts (Figure 6E). Spatial transcriptomics also demonstrated a direct association between *VPS25* and *PVR* expression in tumor regions (Figure S9D and Figure 6F). Importantly, silencing *VPS25* in CAL27 cells led to decreased PVR expression at both transcriptional and translational levels (Figure 6G,H). These findings suggest that *VPS25*^high^ cancer cells contribute to an immunosuppressive TME by modulating T cell cytotoxicity and promoting Treg function, potentially through the regulation of the PVR-TIGIT signaling axis.

### 3.7. VPS25 Predicts Immunotherapy Response in HNSCC Patients

**Figure 7:**
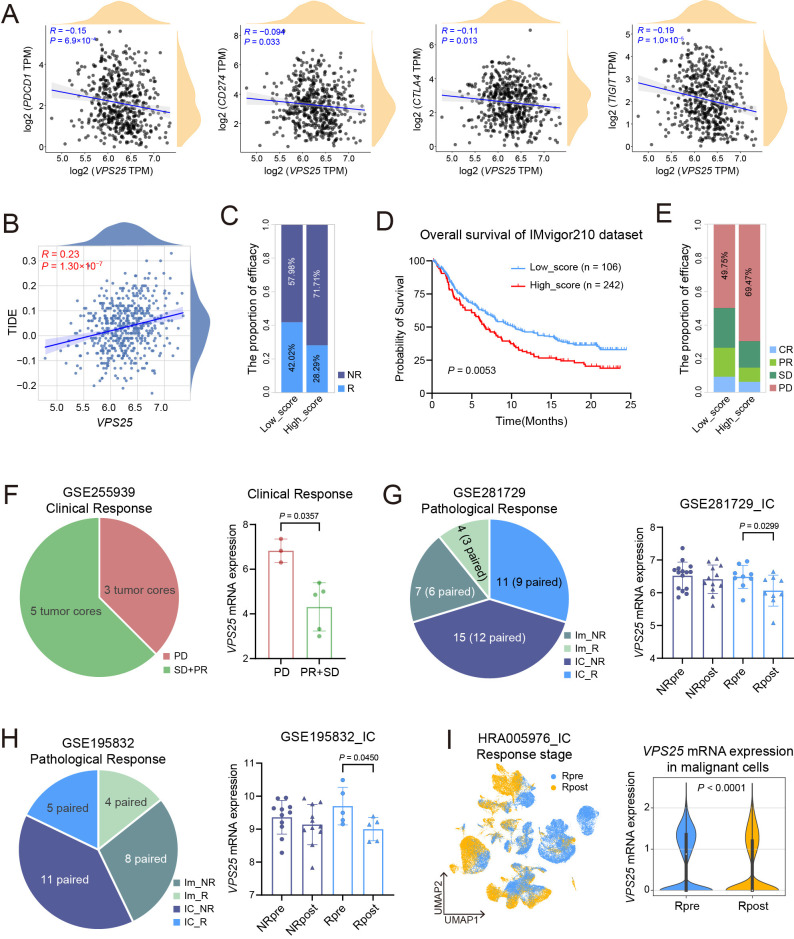
Analysis of the relationship between *VPS25* and immunotherapy efficacy. (**A**) Correlation analyses between *VPS25* expression and immune checkpoint genes, including *PDCD1*, *CD274*, *CTLA4*, and *TIGIT*. (**B**) Correlation analysis between TIDE scores and *VPS25* mRNA expression levels. (**C**) Percentages of immune checkpoint blockade (ICB) responders in the *VPS25*^low^ score cohort compared to the *VPS25*^high^ score cohort. (**D**) Overall survival curves based on the *VPS25*^high^ cancer cell classifier (high vs. low) in the IMvigor210 dataset. (**E**) Comparison of response rates to anti-PD-L1 treatment between low-score cohort and high-score cohort in the IMvigor210 study. (**F**) Pie chart summarizing clinical efficacy information from the GSE255939 dataset, alongside statistical analysis of differences in *VPS25* expression levels among groups with varying treatment responses. (**G**,**H**) Pie charts depicting treatment and pathological response information from the GSE281729 (**G**) and GSE195832 (**H**) datasets, coupled with statistical analyses of *VPS25* expression level changes before and after combination immunotherapy across different pathological response groups. (**I**) UMAP visualization of scRNA-seq integration for tumor samples from responders to immunotherapy combined with chemotherapy in the HRA005976 dataset, accompanied by violin plots showing alterations in *VPS25* expression profiles in cancer cells after treatment. NR: non-response, R: response, CR: complete response, PR: partial response, SD: stable disease, PD: progressive disease, Im: immunotherapy, IC: immunotherapy combined therapy.

To evaluate the role of VPS25 in tumor immune checkpoint blockade (ICB) therapy, we investigated its impact on ICB efficacy. We first examined the correlation between *VPS25* expression and immune checkpoint genes. *VPS25* levels were inversely correlated with *PDCD1*, *CD274*, *CTLA4*, and *TIGIT*, and positive correlated with *CD276*, *CD70*, *HHLA2*, and *VTCN1* (Figure 7A and Figure S10A). Analysis via the TIDE algorithm revealed a direct relationship between *VPS25* expression and TIDE scores in predicting HNSCC patients’ ICB responsiveness (Figure 7B). Higher TIDE scores, indicative of stronger immune evasion, were associated with a decreased likelihood of response to ICB therapy. When stratifying patients based on TIDE score (with scores < 0 as responders), we observed that patients with *VPS25*^high^ HNSCC tumors exhibited lower response rates to ICBs (Figure 7C).

We further expanded our analysis using the IMvigor210 dataset, which includes clinical and RNA-sequencing data from 348 urothelial carcinoma patients treated with Atezolizumab. Higher *VPS25* levels were linked to poorer long-term survival outcomes in patients who had not received immunotherapy (Figure S10B). We then categorized patients based on *VPS25* and *KRT5* (tumor cell marker gene) expression into high-score and low-score groups. Survival and efficacy analyses indicated that patients in the low-score group had better overall survival and improved response to immunotherapy (Figure 7D,E). Similar trends were observed in the HNSCC dataset GSE255939, where patients with low scores demonstrated favorable clinical outcomes following immunotherapy (Figure 7F).

With the increasing use of combination therapies and neoadjuvant immunotherapy, more precise pathological assessments of treatment efficacy have become essential [48,49,50,51,52]. We analyzed two HNSCC neoadjuvant immunotherapy datasets: GSE281729 (anti-PD-1 monotherapy vs. anti-PD-1 + IDO inhibitor) and GSE195832 (anti-PD-1 monotherapy vs. anti-PD-1 + Tadalafil). Pathological evaluations showed no significant change in *VPS25* expression within the monotherapy group (Figure S10C,D), whereas *VPS25* expression significantly decreased in patients who responded to combination therapies (Figure 7G,H). Additionally, single-cell analysis of the HRA005976 dataset, which includes patients receiving anti-PD-1 immunotherapy combined with TPF chemotherapy, revealed a significant reduction in *VPS25* expression in malignant cells from responders after treatment (Figure 7I). This observation indirectly highlights the limitations of current single-agent anti-PD-1 therapy in HNSCC and implies that *VPS25* could function as a potential molecular biomarker for predicting response to combination therapy.

## 4. Discussion

Although previous studies have indicated a tumor-suppressive role for VPS25 [12,53], our current research reveals that VPS25 is significantly upregulated in HNSCC cells and correlates with poor clinical outcomes. This finding suggests a tumor-supportive function for VPS25 in HNSCC. Further bioinformatic analyses and experimental studies demonstrated that VPS25 enhances tumor growth and migration, potentially through the regulation of DNA repair mechanisms. IHC and spatial transcriptomic analyses revealed reduced immune cell infiltration in HNSCC with high VPS25 expression, which may contribute to increased expression of PVR in VPS25^high^ cells. Additionally, we identified *VPS25* as a promising predictive biomarker for immunotherapy. Our findings underscore the multifaceted involvement of VPS25 in tumor progression, highlighting the need for further in-depth investigations into its mechanisms of action.

The crucial role of the ESCRT complex in tumor progression is largely contingent upon its ability to regulate the malignant behaviors of cancer cells. Given its significant function in membrane trafficking, the ESCRT complex influences the activities of receptor tyrosine kinases (RTKs) in cancer cells. For instance, dysfunction of VPS36 has been shown to prevent EGFR degradation, leading to sustained EGFR accumulation and prolonged activation of downstream signaling pathways that drive cell proliferation, survival, migration, and invasion [54]. Furthermore, STAM2 significantly influences the expression of MMP2/MMP9, as well as the phosphorylation of JAK2/STAT3 in cancer cells, thereby enhancing tumor malignancy [55]. Additionally, the interaction between ESCRT subunits and the cytoskeleton is critical for cancer cell migration. For example, TSG101 deficiency in glioma cells impairs actin cytoskeletal organization, resulting in diminished migratory and invasive potential [56]. ESCRT subunits have also been linked to the modulation of cell cycle processes and therapeutic resistance. Elevated STAMBP in pancreatic cancer has been found to interact with E2F1, preventing its degradation and promoting the PDK1-mediated Warburg effect, which contributes to chemotherapy resistance [57]. In this study, we observed that *VPS25* is highly expressed in cancer cell-enriched genes related to DNA repair, E2F targets, and G2M checkpoints, highlighting its essential role in proliferation and cell cycle progression. Moreover, the knockdown of *VPS25* in CAL27 cells resulted in decreased proliferation and migration. These findings align with previous studies indicating that VPS25 promotes tumor progression by modulating the cell cycle and apoptosis, particularly through JAK-STAT signaling [58].

The ESCRT also plays a pivotal role in tumor progression by modulating processes such as membrane receptor recycling, exosome secretion, and membrane repair, thereby influencing the TME. Previous studies have reported that phosphorylation of HRS, a component of the ESCRT-0 complex, promotes the release of immunosuppressive exosomes and inhibits CD8^+^ T cell tumor infiltration [59]. Additionally, our prior investigation established HRS as a critical regulator in immune evasion through the activation of proteostasis-associated interferon pathways [60], and HRS depletion sensitizes melanoma and HNSCC to anti-PD-1 therapy [61]. Furthermore, CHMP2A has been shown to induce apoptosis in NK cells, thereby reducing their anti-tumor activity through the secretion of EVs expressing MICA/B and TRAIL [18]. This was confirmed in an mCHMP2A knockout 4MOSC1 immunocompetent mouse model, where the absence of CHMP2A led to increased infiltration of CD4^+^ and CD8^+^ T cells [62]. Moreover, the ESCRT machinery is crucial for repairing membrane lesions by being recruited to damaged areas, preventing excessive cell death, and modulating the pyroptotic response [63]. Similarly, during CTL-mediated cytotoxicity, ESCRT proteins are recruited to perforin-induced membrane lesions in target cells, facilitating membrane repair and limiting granzyme entry into the cytosol [17]. This repair mechanism is vital for tumor cells to resist immune-mediated lysis. Our investigation revealed an inverse correlation between VPS25 expression levels and tumor infiltration by immunostimulatory cells (CD8^+^ T cells, NK cells, B cells, and DCs), whereas a positive association was observed with immunosuppressive cell populations (MDSCs and M2-polarized macrophages). Within the TME, VPS25^high^ cancer cells were positioned farther from effector immune cells. Mechanistically, we observed increased PVR expression in VPS25^high^ cancer cells, and knockdown of *VPS25* reduced PVR expression, suggesting that VPS25 may mediate immune evasion through the PVR-TIGIT axis.

The dual role of VPS25 in HNSCC progression positions it as both a viable predictive biomarker for immunotherapy response and a promising molecular target for therapeutic intervention. VPS25 expression patterns may enable refined patient classification, supporting the implementation of precision oncology strategies customized according to individual molecular and immunological profiles, especially for ICB-based treatment regimens. Additionally, targeting VPS25 may simultaneously disrupt tumor progression and enhance anti-tumor immunity.

However, this study has limitations. The precise molecular mechanisms through which VPS25 regulates immune cell recruitment and function remain to be fully elucidated. Functional studies examining the direct interactions between VPS25 and immune checkpoint molecules, both in vitro and in vivo, are necessary to clarify these pathways. Furthermore, our findings regarding immunotherapy response prediction are based on retrospective analyses, which may not completely reflect clinical realities. Prospective clinical trials are essential to validate the utility of VPS25 as a biomarker and therapeutic target. Future research should focus on experimentally validating the molecular mechanisms by which VPS25 modulates tumor immunology. Additionally, it would be valuable to investigate VPS25-targeting therapies, alone or in combination with ICBs in pre-clinical models. Exploring the role of VPS25 in other cancer types and its broader implications within the ESCRT pathway may provide a more comprehensive understanding of its functions in tumor biology.

## 5. Conclusions

In summary, our findings underscore the pivotal involvement of VPS25 in the progression and immunosuppressive modulation of HNSCC. VPS25 overexpression in HNSCC is linked to poor prognosis, likely by promoting an immunosuppressive microenvironment through PVR upregulation, which activates the TIGIT pathway. It serves as both a prognostic marker and a potential predictor of immunotherapy response, offering new opportunities for targeted treatment.

## Data Availability

Publicly available datasets can be found in the TCGA database and the GEO database. Additional datasets and materials generated during this research can be obtained from the corresponding author through formal request, subject to reasonable justification.

## Supplementary Materials

The following supporting information can be downloaded at https://www.mdpi.com/article/10.3390/biom15030323/s1: Table S1: The clinicopathological features of HNSCC patients; Figure S1. Comparative analysis of ESCRT subunit expression and prognostic implications in HNSCC; Figure S2. Overall survival analysis of significantly overexpressed ESCRT subunits in HNSCC tumor tissues; Figure S3. The relationship between VPS25 protein expression levels in tumor tissues and clinicopathological features of HNSCC; Figure S4. Composition analysis of the HNSCC tumor microenvironment; Figure S5. RNA sequencing of *VPS25* KD CAL27 cells; Figure S6. scRNA-seq data annotation for spatial transcriptome deconvolution; Figures S7 and S8. Spatial analysis of *VPS25*^high^ cancer cells and tumor-infiltrating immune cells; Figure S9. scRNA-seq and ST analysis of T cell subsets in HNSCC; Figure S10. Relationship between *VPS25* expression and immunotherapy response; Figure S11: Original Western blot image of Figure 3A; Figure S12: Original Western blot image of Figure 6G.

## Abbreviations

The following abbreviations are used in this manuscript:

HNSCC	Head and neck squamous cell carcinoma
ESCRT	Endosomal sorting complex required for transport
TCGA	The Cancer Genome Atlas
VPS25	Vacuolar Protein Sorting 25 Homolog
HR	Hazard ratio
NOM	Normal oral mucosa
OS	Overall survival
DFS	Disease-free survival
TMAs	Tissue microarrays
IHC	Immunohistochemistry
TME	Tumor microenvironment
scRNA-seq	Single-cell RNA sequencing
CNV	Copy number variation
UMAP	Uniform manifold approximation and projection
pEMT	Partial epithelial–mesenchymal transition
ST	Spatial transcriptomics
DEG	Differentially expressed gene
GSEA	Gene set enrichment analysis
NES	Normalized enrichment score
FDR	False discovery rate
KEGG	Kyoto Encyclopedia of Genes and Genomes
GOBP	Gene ontology biological process
RSS	Regulon specificity score
TILs	Tumor-infiltrating lymphocytes
TIMs	Tumor-infiltrating myeloid cells
TAMs	Tumor-associated macrophages
MDSCs	Myeloid-derived suppressor cells
Tregs	Regulatory T cells
PVR	Poliovirus receptor
TIDE	Tumor immune dysfunction and exclusion
ICB	immune checkpoint blockade
Im	Immunotherapy
IC	Immunotherapy combined therapy
R	Response
NR	Non-response
CR	Complete response
PR	Partial response
SD	Stable disease
PD	Progressive disease
