# Supplementary material for: VPS25 Promotes an Immunosuppressive Microenvironment in Head and Neck Squamous Cell Carcinoma
Source: Biomolecules. 2025 Feb 22;15(3):323. doi: 10.3390/biom15030323 (PMC11940596; doi:10.3390/biom15030323)
Supplement: Supplementary file 1 [file biomolecules-15-00323-s001.zip › Schematic Diagram.pdf]

Schematic diagram for introduction

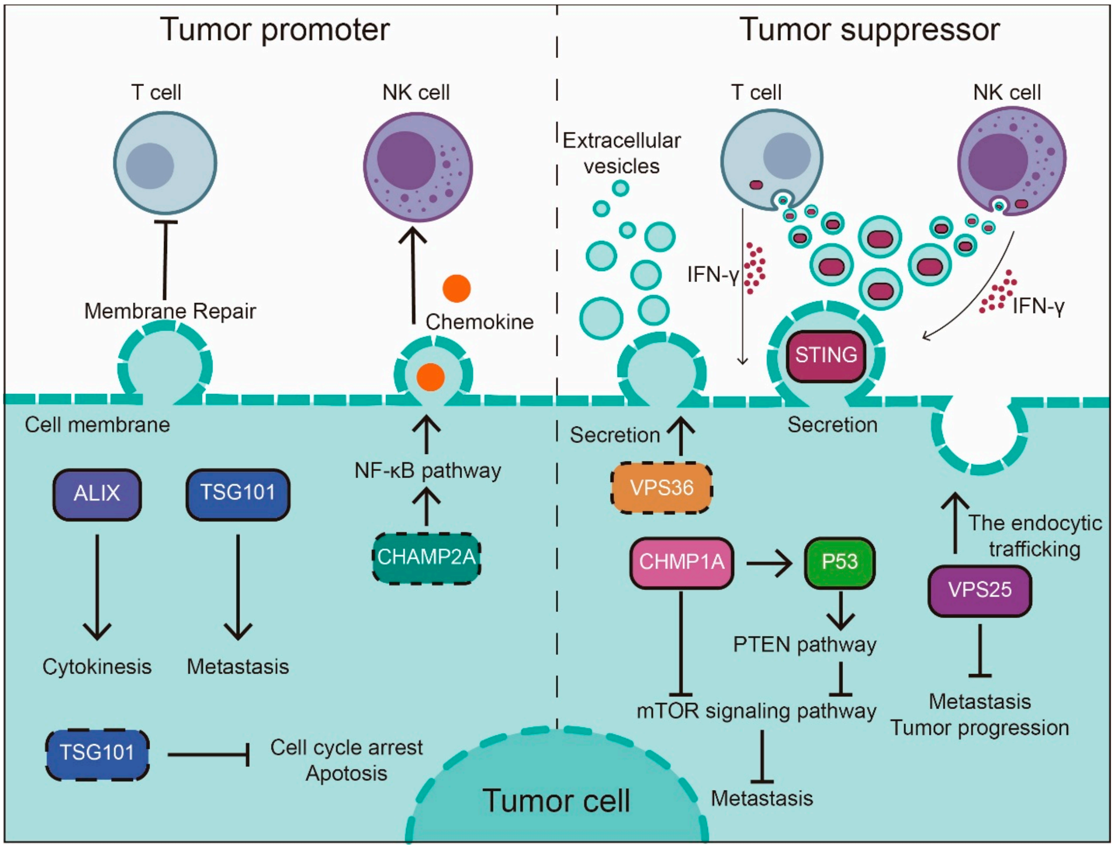

The role of ESCRT complex in tumors.

The dashed boxes in the figure indicate a reduction in the expression of ESCRT-related subunit proteins.

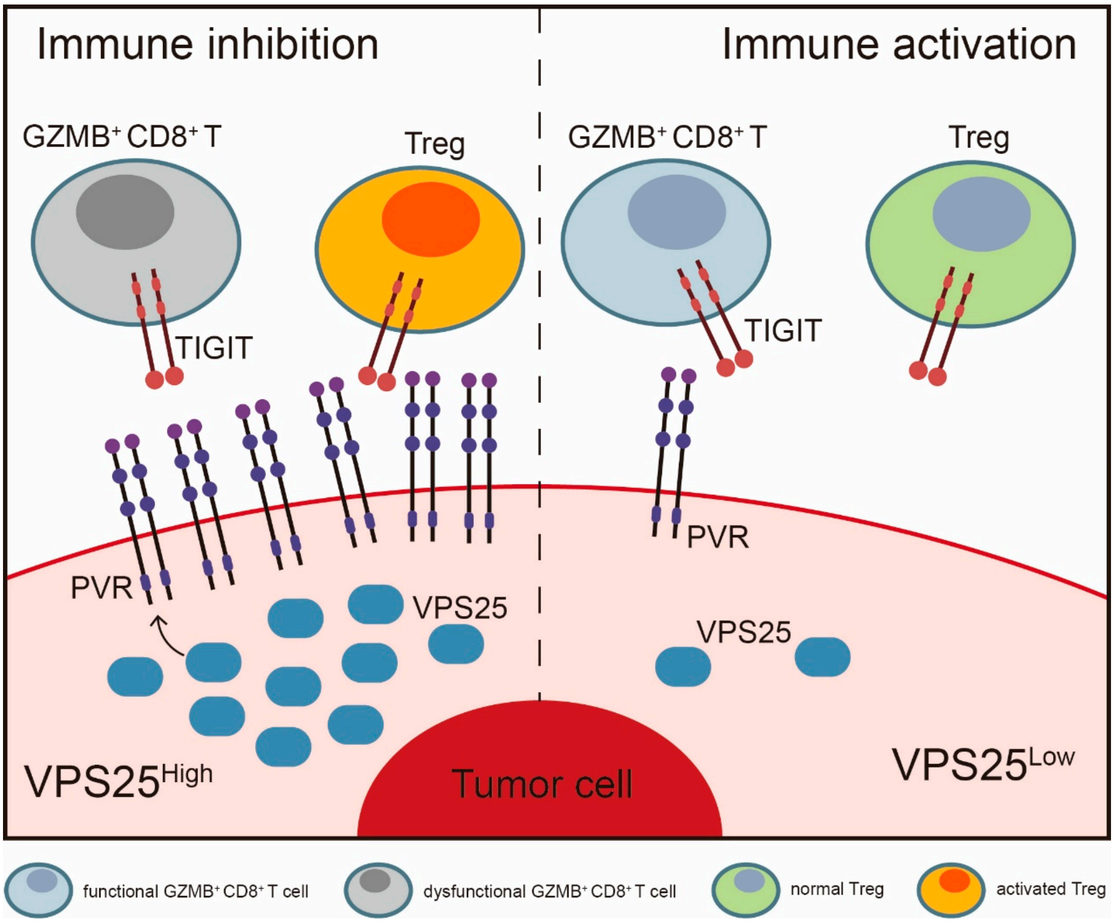

VPS25^{high} tumor cells exert immunosuppressive effects through the PVR-TIGIT signaling axis.
