# Supplementary material for: VPS25 Promotes an Immunosuppressive Microenvironment in Head and Neck Squamous Cell Carcinoma
Source: Biomolecules. 2025 Feb 22;15(3):323. doi: 10.3390/biom15030323 (PMC11940596; doi:10.3390/biom15030323)
Supplement: Supplementary file 1 [file biomolecules-15-00323-s001.zip › Supplementary Materials (Table,Figures).pdf]

Supplementary Materials

Supplementary Table

Table S1. The clinicopathological features of HNSCC patients.

No. patient	Gender	Age	TNM	Clinical stage	Pathological stage
1	Female	64	T2N0M0	II	I
2	Male	58	T2N0M0	II	I
3	Male	46	T2M0N0	II	I
4	Male	38	T2N0M0	II	I
5	Male	55	T2N0M0	II	I
6	Male	44	T1N0M0	I	I
7	Female	44	T2N0M0	II	I
8	Male	43	T2N0M1	IV	I
9	Male	70	T2N0M0	II	I
10	Male	53	T2N0Mx	NA	I
11	Male	54	T4N0M0	IV	I
12	Male	57	T2N0M0	II	I
13	Male	65	T2N1M0	III	I
14	Male	46	TxN3M0	IV	I
15	Male	60	T3N0M0	III	I
16	Male	60	T2N0M0	II	I
17	Female	37	T2N0M0	II	II
18	Male	62	T3N2M0	IV	III
19	Male	46	T3N0M0	III	I
20	Male	69	T2N1M0	III	I
21	Male	60	T4N1M0	IV	I
22	Female	55	T2N0M0	II	I
23	Male	73	T3N2M0	IV	I
24	Male	64	T2N1M0	III	I
25	Male	56	T4aN2aM0	IV	III
26	Male	44	T2N2M0	IV	I
27	Male	58	T2N0M0	II	I
28	Male	48	T4aN1M0	IV	III
29	Male	44	T2N0M0	II	I
30	Male	50	T3N0M0	III	I
31	Male	46	T2N0M0	II	II
32	Male	43	T3N0M0	III	I
33	Male	60	T2N0M0	II	I
34	Male	41	T4aN2M0	IV	I
35	Male	59	T2N1M0	III	III
36	Female	64	T1N0M0	I	I
37	Male	53	T2N0M0	II	I
38	Female	43	T2N0M0	II	I
39	Male	53	T2N0M0	II	II
40	Male	69	T3N0M0	III	I
41	Male	64	T3N0M0	III	II
42	Male	60	T4N2bM0	IV	II
43	Male	56	T4N1M0	IV	II
44	Female	55	T2N0M0	II	II

45	Male	55	T3N1M0	III	II
46	Male	62	T3N0M0	III	II
47	Male	62	T3N1M0	III	II
48	Male	57	T4N2cM0	IV	II
49	Male	60	T2N0M0	II	II
50	Male	31	T2N1M0	III	III
51	Male	76	T2N2M0	IV	II
52	Female	70	T3N2M0	IV	II
53	Male	68	T1N0M0	I	I
54	Male	54	T4N2M0	IV	I
55	Male	54	T4N0M0	IV	II
56	Male	46	T4N0M0	IV	II
57	Male	71	T3N0M0	III	I
58	Male	67	T3N0M0	III	II
59	Female	55	T2N2M0	IV	II
60	Male	60	T4N1M0	IV	II
61	Female	77	T3N0M0	III	II
62	Male	63	T2N0M0	II	I
63	Male	74	T3N2CM0	IV	II
64	Female	77	T2N0M0	II	I
65	Male	58	T4N0M0	IV	I
66	Male	31	T3N1M0	III	II
67	Male	36	T2N1M0	III	III
68	Female	68	T2N0M0	II	I
69	Male	38	T4N2M0	IV	II
70	Male	66	T3N1M0	III	II
71	Male	41	T2N1M0	III	II
72	Male	58	T3N0M0	III	II
73	Male	55	T2N0M0	II	II
74	Male	53	T3N1M0	III	II
75	Male	64	T3N0M0	III	II
76	Male	69	T2N0M0	II	II
77	Male	61	T2N0M0	II	II
78	Female	71	T2N0M0	II	I
79	Male	63	T2N2M0	IV	II
80	Male	70	T3N3M0	IV	II
81	Male	68	T2N1M0	III	II
82	Male	31	T3N0M0	III	III
83	Female	66	T4N2M0	IV	I
84	Male	65	T2N0M0	II	II
85	Male	63	T2N2bM0	IV	III
86	Male	64	T4N0M0	IV	II
87	Female	70	T4N1M0	IV	II
88	Male	51	T4N1M0	IV	II
89	Male	64	T2N0M0	II	I
90	Male	82	T4N1M0	IV	II
91	Male	41	T3N1M0	III	II
92	Male	48	T4N2M0	IV	II
93	Male	62	T3N0M0	III	III
94	Female	64	T4N0M0	IV	I
95	Female	66	T2N1M0	III	II

96	Male	75	T3N1M0	III	II
97	Female	60	T2N0M0	II	II
98	Male	65	T2N0M0	II	II
99	Male	39	T2N1M0	III	II
100	Male	58	T4N0M0	IV	II
101	Female	70	T2N3M0	IV	II
102	Male	65	T3N3M0	IV	III
103	Male	50	T4N0M0	IV	II
104	Male	48	T3N2M0	IV	III
105	Male	82	T4N0M0	IV	III
106	Female	77	T1N0M0	I	III
107	Male	64	T4N1M0	IV	II
108	Female	74	T4N0M0	IV	NA
109	Male	56	T3N0M0	III	II
110	Male	75	T2N0M0	II	II
111	Male	77	T2N0M0	II	III
112	Male	29	T2N0M0	II	II
113	Male	39	T2N0M0	II	II
114	Male	66	T4N2M0	IV	II
115	Male	63	T3N0M0	III	NA
116	Female	64	T2N0M0	II	II
117	Female	49	T2N0M0	II	II
118	Male	62	T3N0M0	III	II
119	Male	51	T4N1M0	IV	II
120	Male	63	T2N0M0	II	II
121	Male	48	T4N2M0	IV	III
122	Male	38	T1N0M0	I	II
123	Male	47	T3N0M0	III	II
124	Male	56	T2N0M0	II	II
125	Female	55	T4N3M0	IV	II
126	Male	50	T2N0M0	II	II
127	Male	72	T2N0M0	II	II
128	Male	53	T3N0M0	III	II
129	Male	52	T2N3M0	IV	III
130	Female	59	T2N0M0	II	I
131	Male	58	T4N2M0	IV	I
132	Male	53	T1M0N0	I	III
133	Female	56	T4aN1Mx	NA	I
134	Male	41	T1M0N0	I	II
135	Male	51	T2N0M0	II	II
136	Male	44	T3N0M0	III	I
137	Male	59	T1N0M0	I	II
138	Male	47	T2N0M0	II	I

Supplementary Figures

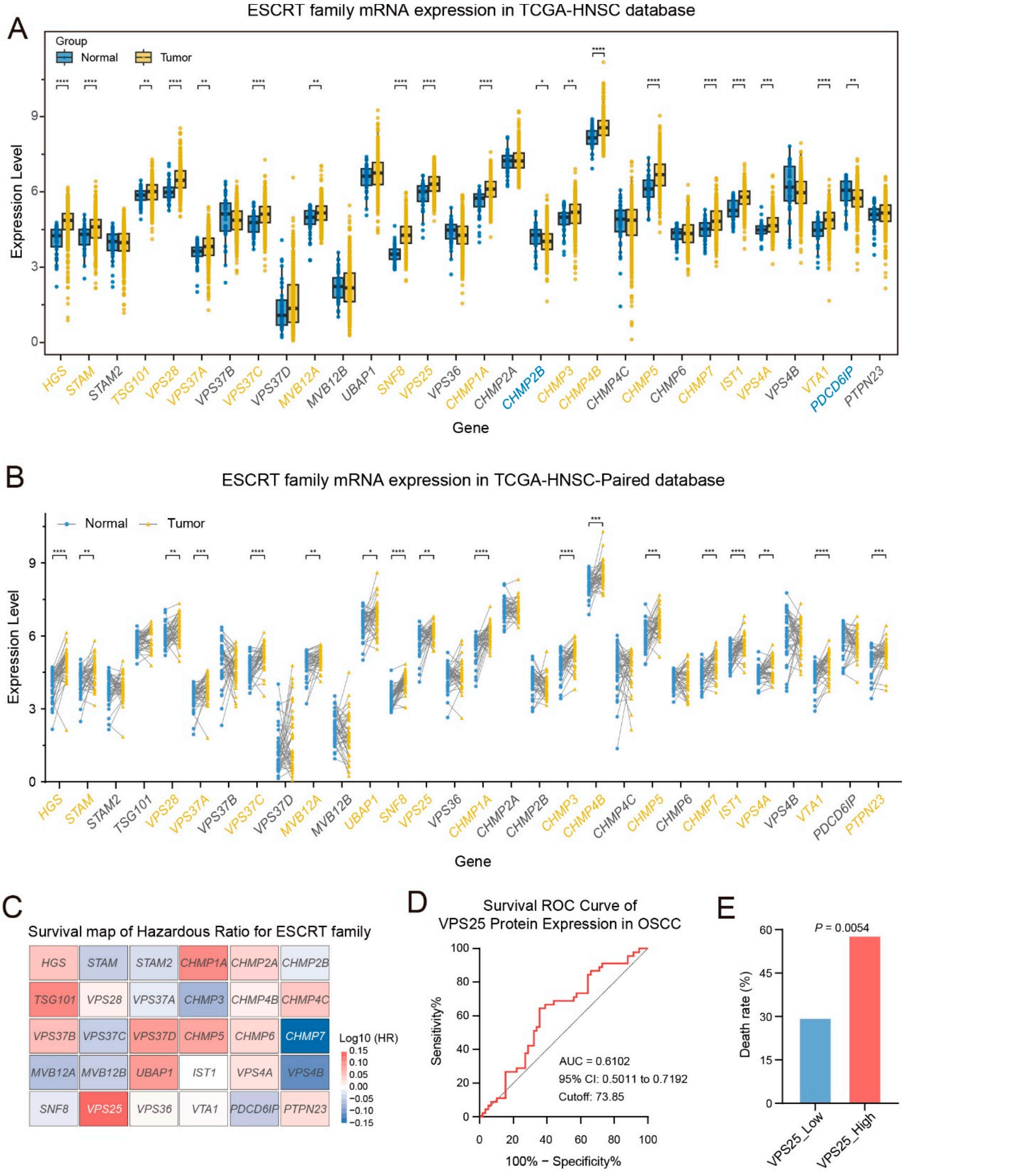

Figure S1. Comparative analysis of ESCRT subunit expression and prognostic implications in HNSCC. (A) Unpaired t-test comparing mRNA expression levels of ESCRT subunits between normal tissues and HNSCC tumor tissues. (B) Paired t-test evaluating mRNA expression levels of ESCRT subunits in normal tissues and tumor tissues derived from the same patients. (C) Heatmap of hazard ratios for overall survival associated with the expression of ESCRT family members. (D) Receiver operating characteristic (ROC) curve assessing the prognostic value of VPS25, with an area under the curve (AUC) of 0.6102. (E) Death rate analysis for HNSCC patients with high (n = 50) and low (n = 54) VPS25 expression levels. * $P < 0.05$, ** $P < 0.01$, *** $P < 0.001$, **** $P < 0.0001$.

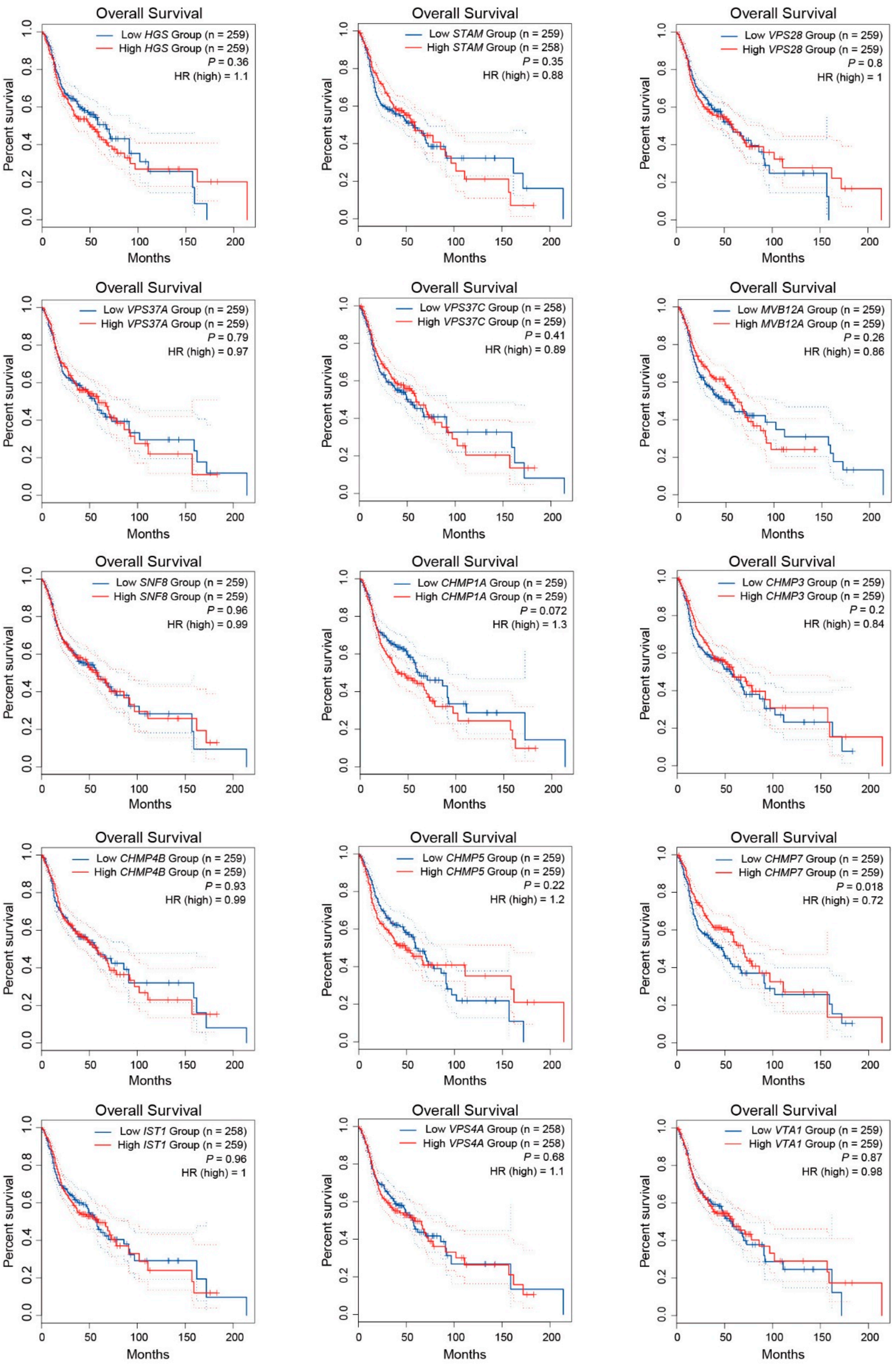

Figure S2. Overall survival analysis of significantly overexpressed ESCRT subunits in HNSCC tumor tissues.

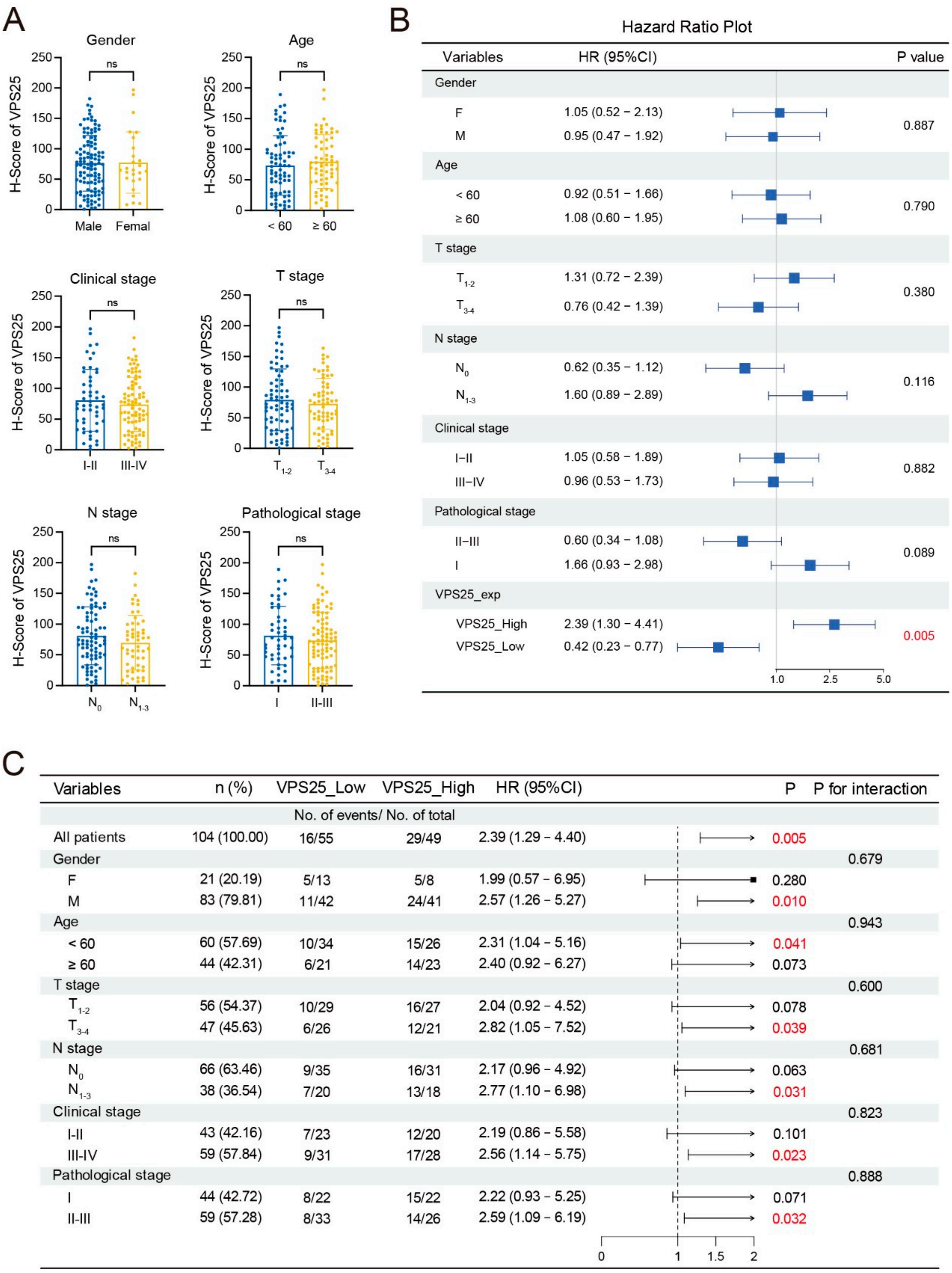

Figure S3. The relationship between VPS25 protein expression levels in tumor tissues and clinicopathological features of HNSCC. (A) Comparison of VPS25 H-scores among HNSCC patients with different clinicopathological characteristics. Data are presented as means ± SD. (B) Cox regression analysis forest plot illustrating the hazard ratios (HRs) and 95% confidence intervals (CIs) for overall survival in HNSCC patients, based on VPS25 protein expression levels and clinicopathological features. (C) Subgroup Cox regression analysis stratified by clinicopathological characteristics, showing HRs for overall survival in patients with high and low VPS25 protein expression.

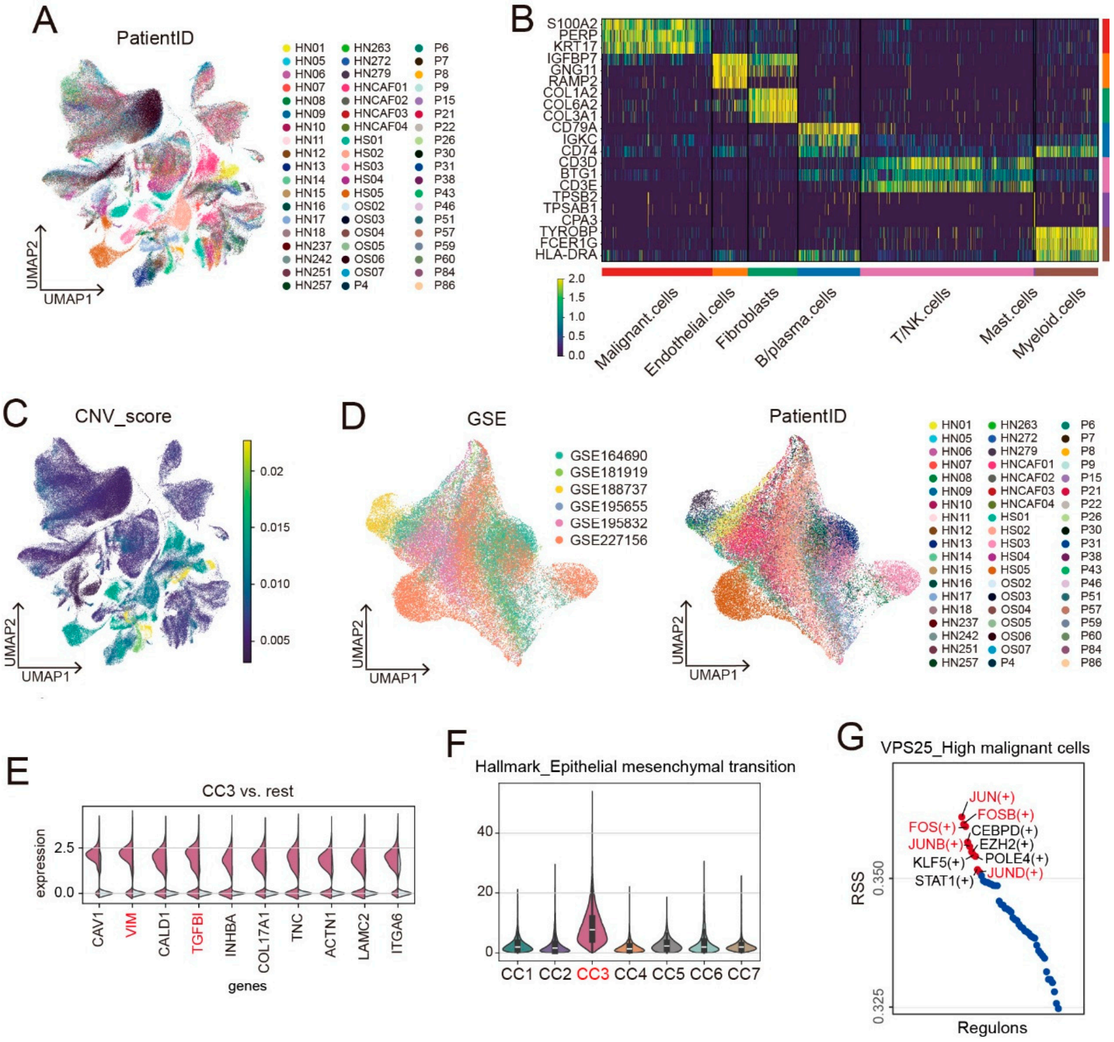

Figure S4. Composition analysis of the HNSCC tumor microenvironment. (A) UMAP embedding displaying patientIDs (n = 57) from 6 HNSCC scRNA-seq databases. (B) Heatmap showing marker genes for distinct cell types in the HNSCC tumor microenvironment. (C) UMAP visualization of copy number variation (CNV) scores across different cell types in the HNSCC tumor microenvironment. (D) UMAP embedding showing the GSE dataset sources and patient origins of all cancer cells. (E) Top 10 marker genes of the CC3 cancer cell sub-cluster. (F) Function enrichment analysis of epithelial-mesenchymal transition among 7 cancer cells subtypes. (G) Upstream transcriptional regulators of VPS25^{high} cancer cells.

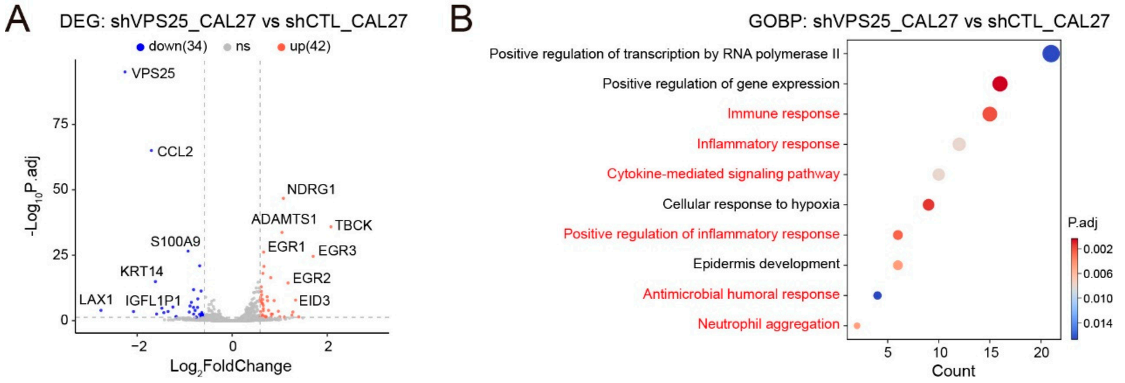

Figure S5. RNA sequencing of VPS25 KD CAL27 cells. (A) Volcano plot of differentially expressed genes (DEGs). Red dots indicate upregulated genes after VPS25 knockdown and blue dots indicate downregulated genes. The x-axis represents log2 of fold change and the y-axis represents -log10 of adjusted p value. (B) Gene ontology biological process (GOBP) enrichment analysis of upregulated DEGs in VPS25 KD CAL27 cells. The y-axis represents the GO terms and the x-axis represents the number of target genes.

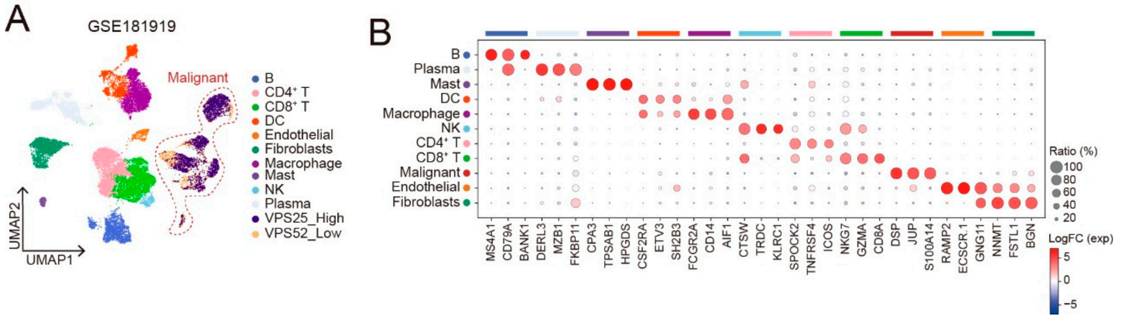

Figure S6. scRNA-seq data annotation for spatial transcriptome deconvolution. (A,B) Subclassification of tumor microenvironment cell populations (A) and marker gene dotplot (B) for each population in the HNSCC scRNA-seq dataset GSE181919.

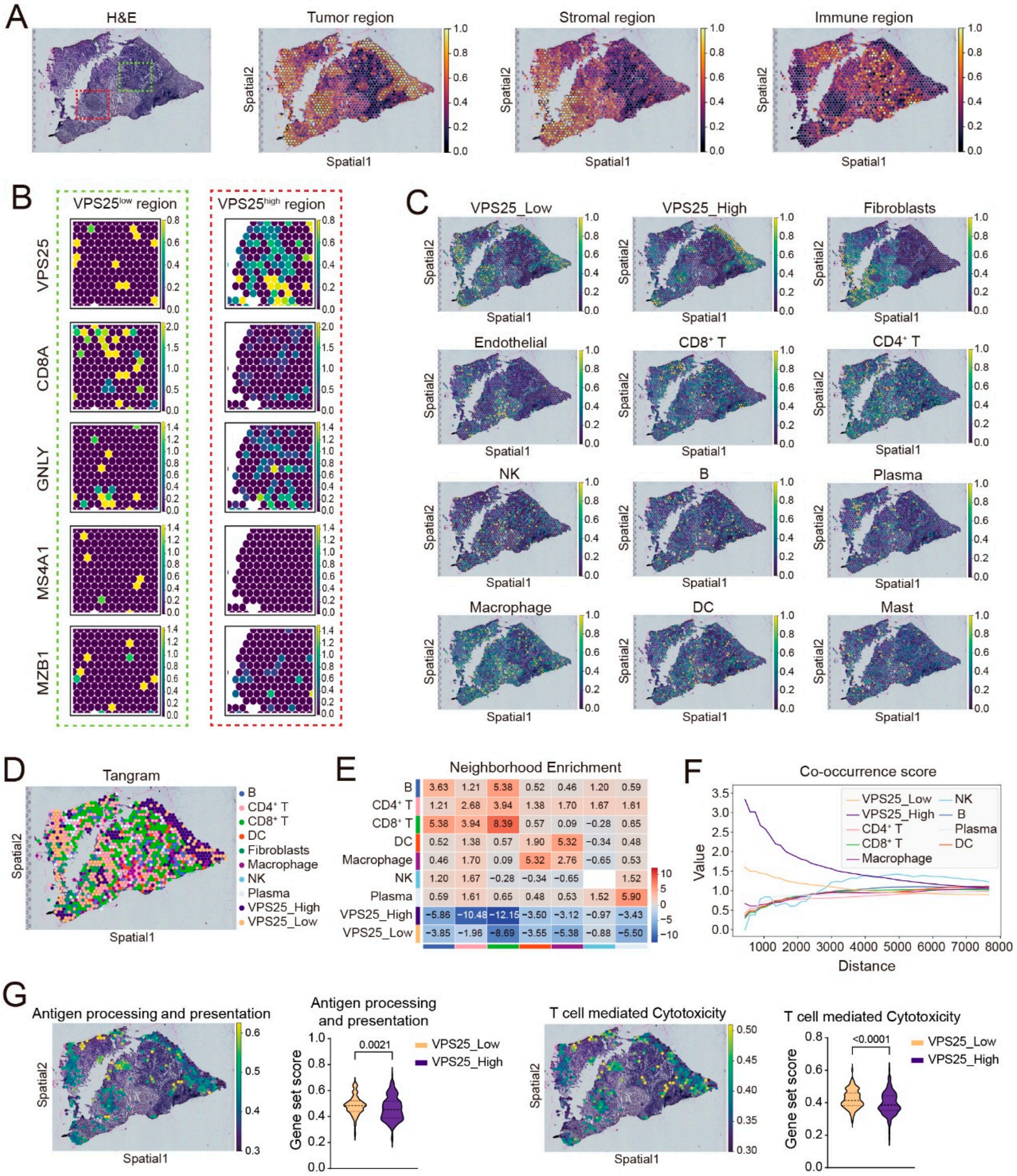

Figure S7. Spatial analysis of VPS25^{high} cancer cells and tumor-infiltrating immune cells. (A) Hematoxylin and eosin (HE) staining of a representative HNSCC tumor tissue with spatial transcriptomics data, showing delineation into tumor, stromal, and immune-infiltrated regions based on marker gene expression. (B) UMAP visualization of expression levels and spatial distribution of immune cell markers in the VPS25 high-expression tumor region (red box area in Figure A) and the VPS25 low-expression tumor region (green box area in Figure A). (C) Mapping scRNA-seq annotations to spatial transcriptomics images. (D) UMAP showing dominant cell type distribution based on ranked subpopulation prediction scores in each spatial spot. (E) Neighbor enrichment score heatmap illustrating spatial location relationships among different cell types. (F) Visualization of cluster co-occurrence in spatial dimensions. (G) UMAP plots showing the enrichment of antigen processing and presentation as well as T cell-mediated cytotoxicity gene sets in VPS25^{low} and VPS25^{high} cancer cells spatial spots. Violin plots display statistical differences in enrichment scores.

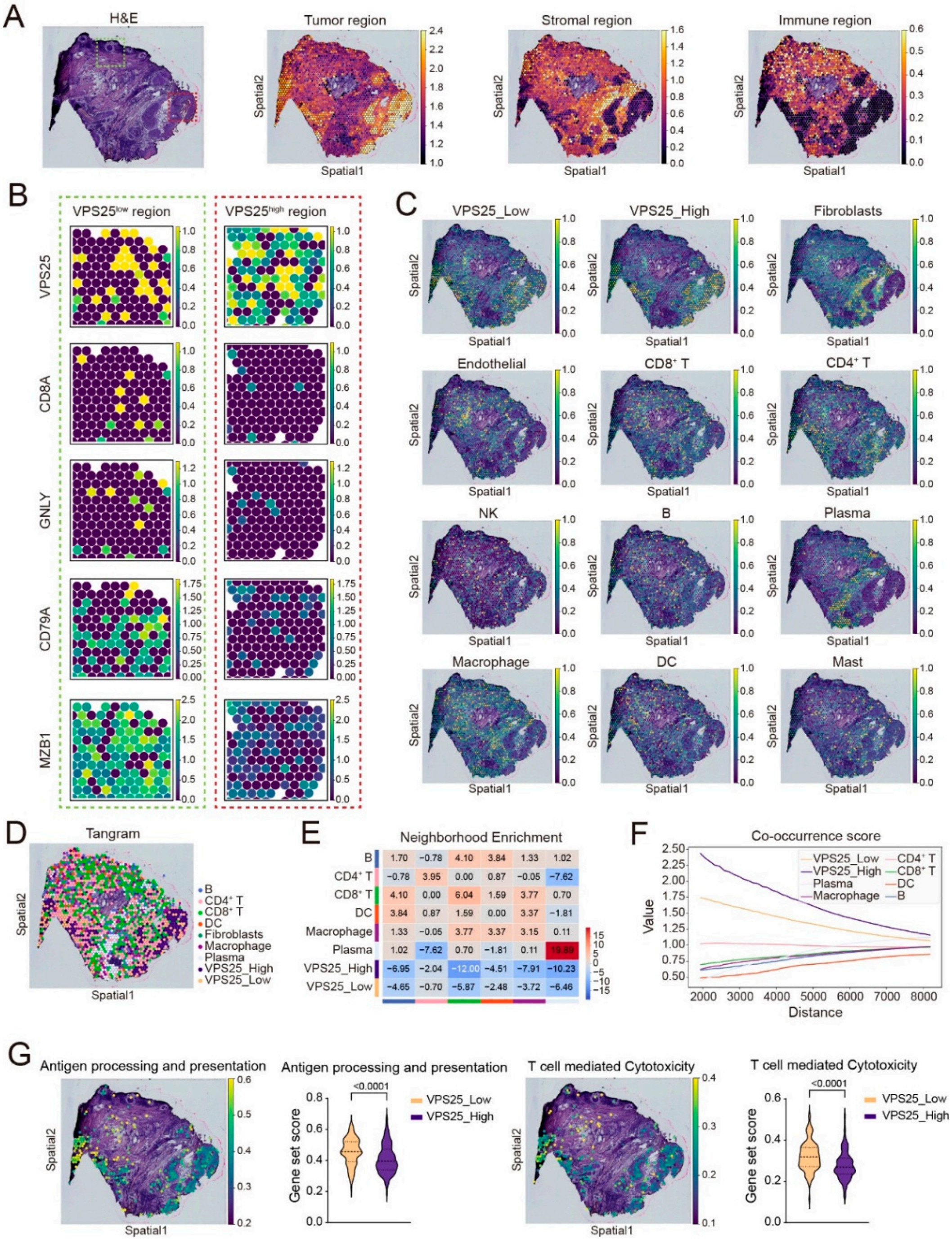

Figure S8. Spatial analysis of VPS25^{high} cancer cells and tumor-infiltrating immune cells. (A) Hematoxylin and eosin (HE) staining of a representative HNSCC tumor tissue with spatial transcriptomics data, showing delineation into tumor, stromal, and immune-infiltrated regions based on marker gene expression. (B) UMAP visualization of expression levels and spatial distribution of immune cell markers in the VPS25 high-expression tumor region (red box area in Figure A) and the VPS25 low-expression tumor region (green box area in Figure A). (C) Mapping scRNA-seq annotations to spatial transcriptomics images. (D) UMAP showing dominant cell type distribution based on ranked subpopulation prediction scores in each spatial spot. (E) Neighbor enrichment score heatmap illustrating spatial location relationships among different cell types. (F) Visualization of cluster co-occurrence in spatial dimensions. (G) UMAP plots showing the enrichment of antigen processing and presentation as well as T cell-mediated cytotoxicity gene sets in VPS25^{low} and VPS25^{high} cancer cells spatial spots. Violin plots display statistical differences in enrichment scores.

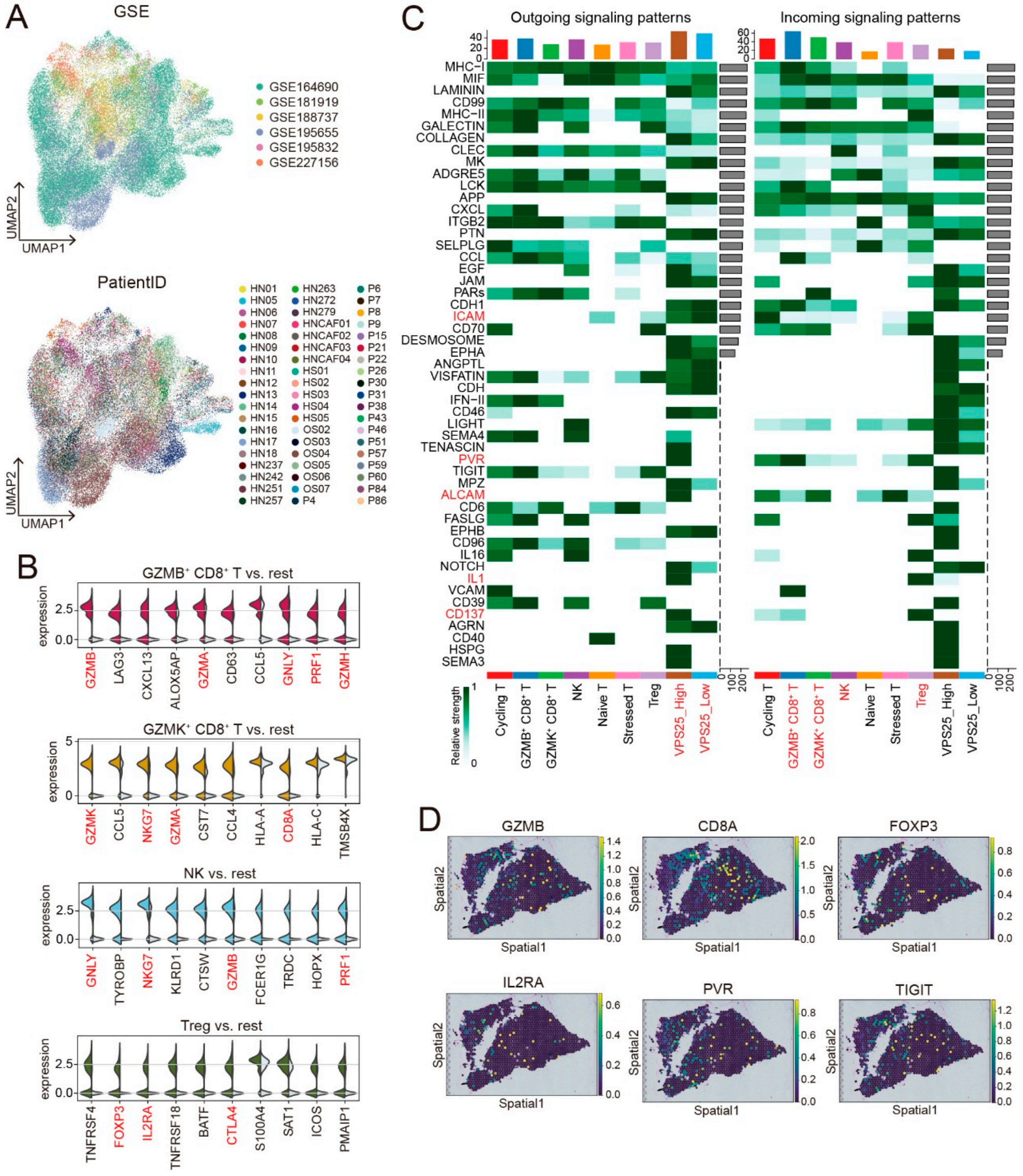

Figure S9. scRNA-seq and ST analysis of T cell subsets in HNSCC. (A) UMAP embedding visualizing tumor-associated T/NK cells, annotated with GSE database resources and patient ID information. (B) Violin plots showing the top 10 marker genes for GZMB⁺ CD8⁺ T cells, GZMK⁺ CD8⁺ T cells, NK cells, and Tregs. (C) The heatmap illustrates the signaling network among tumor cell subpopulations and T/NK cell subpopulations, depicting the senders, receivers, as well as the quantity and intensity of their interactions. (D) Expression and spatial distribution of *GZMB*, *CD8A*, *FOXP3*, *IL2RA*, *PVR*, and *TIGIT* genes in HNSCC tissues.

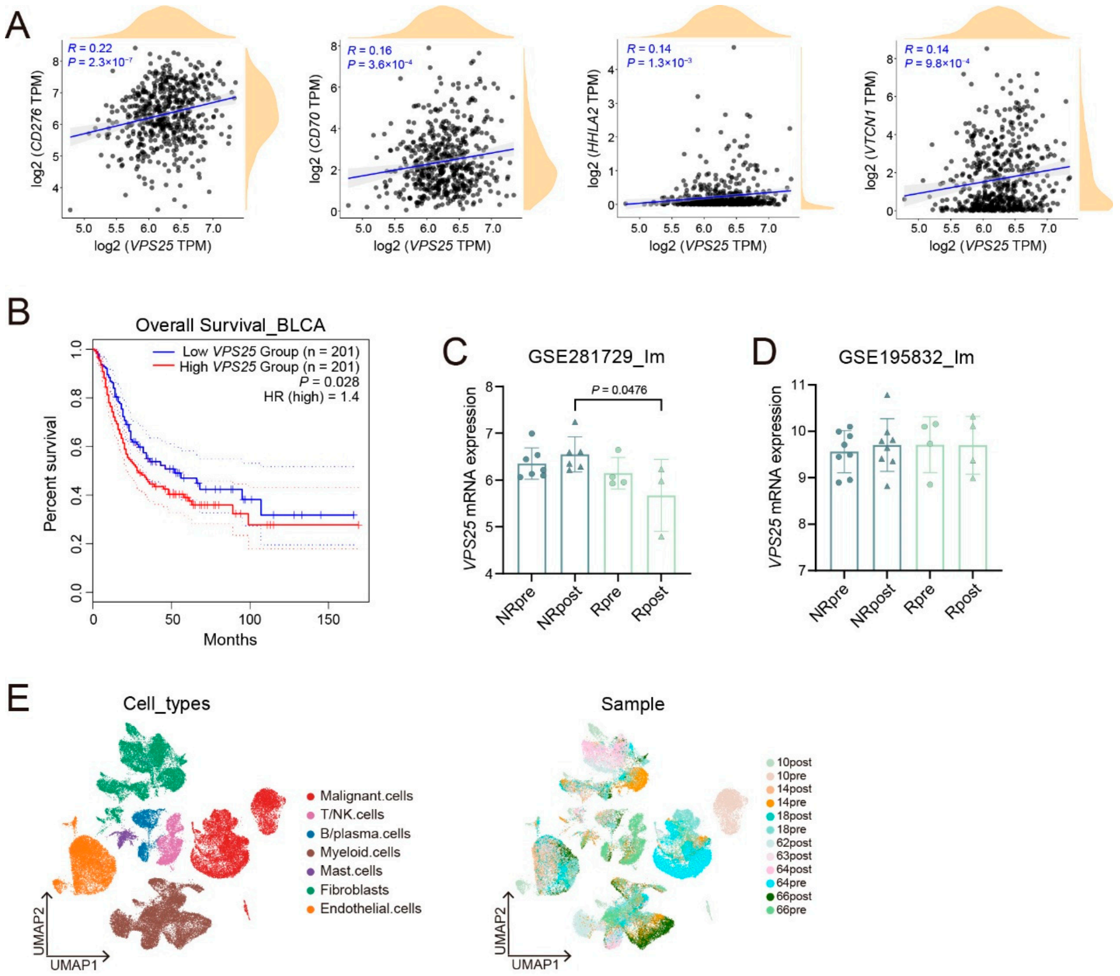

Figure S10. Relationship between VPS25 expression and immunotherapy response. (A) Correlation analyses between VPS25 expression and immune checkpoint genes, including *CD276*, *CD70*, *HHLA2*, and *VTCN1*. (B) Overall survival (OS) curves for bladder cancer (BLCA) patients with high (n = 201) and low (n = 201) VPS25 expression levels. (C, D) Statistical analyses of VPS25 expression level changes before and after monoimmunotherapy across different response groups. (E) UMAP visualization from the HRA005976 dataset showing tumor microenvironment cell annotations and sample source information for responders to immunotherapy combined with chemotherapy. Data are presented as means ± SD (C, D).

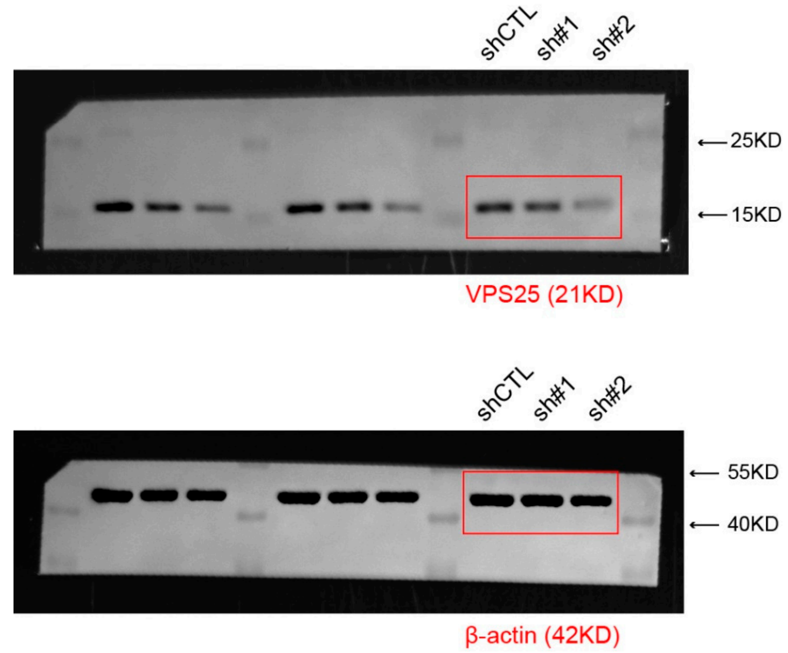

Figure S11. Original Western blot image of Figure 3A.

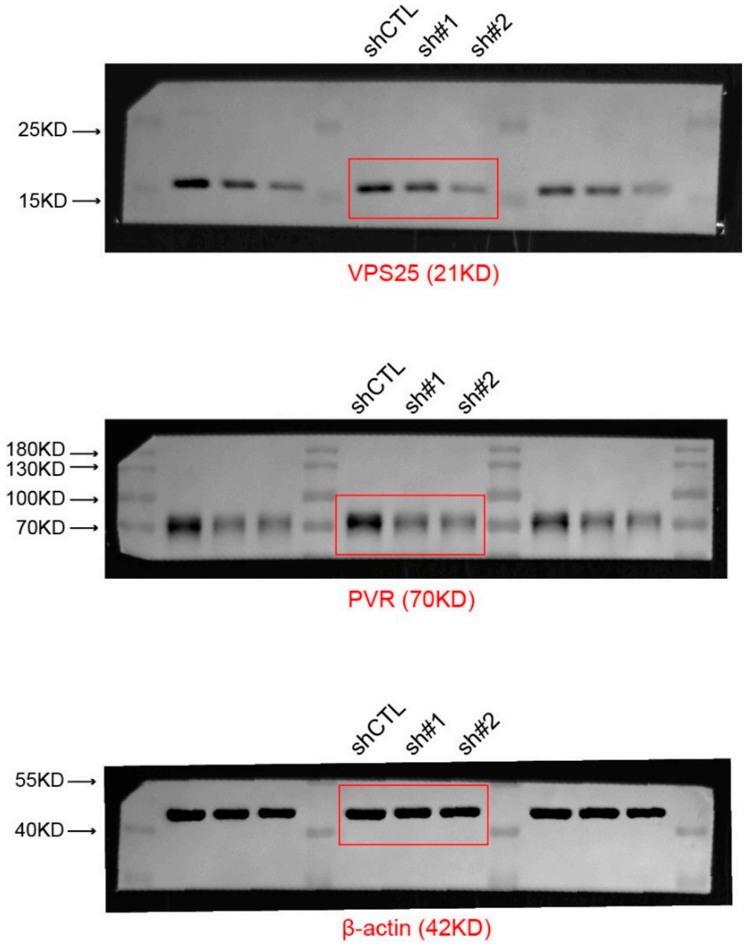

Figure S12. Original Western blot image of Figure 6G.
